# Autophagy controls the hippocampal postsynaptic organization and affects cognition in a mouse model of Fragile X syndrome

**DOI:** 10.1038/s41380-025-03207-6

**Published:** 2025-09-10

**Authors:** Ziyan Zhang, Cameron Keyser, Yaxin Li, Breandan J. Rosolia, Morgan W. Porch, Wen Zhang, Bin Su, Peng Jiang, R. Suzanne Zukin, Jingqi Yan

**Affiliations:** 1 Center for Gene Regulation in Health and Disease, Cleveland State University, Cleveland, OH 44115 USA; 2 Department of Chemistry, Center for Gene Regulation in Health and Disease, Cleveland State University, Cleveland, OH 44115 USA; 3 Dominick P. Purpura Department of Neuroscience, Albert Einstein College of Medicine, Bronx, NY 10461 USA; 4 Department of Psychiatry, Friedman Brain Institute, and Department of Genetics and Genomic Science and Institute for Multiscale Biology, Icahn School of Medicine at Mount Sinai, New York, NY 10029 USA

**Keywords:** Neuroscience, Drug discovery

## Abstract

Dysregulated spine morphology is a common feature in the pathology of many neurodevelopmental and neuropsychiatric disorders. Overabundant immature dendritic spines in the hippocampus are causally related to cognitive deficits of Fragile X syndrome (FXS), the most common form of heritable intellectual disability. Recent findings from us and others indicate autophagy plays important roles in synaptic stability and morphology, and autophagy is downregulated in FXS neurons. However, the mechanism remains unclear. In this study, we identified that activated autophagy degrades the eukaryotic initiation factor 4G1 (eIF4G1) and postsynaptic density protein-95 (PSD-95) in hippocampal neurons of *Fmr1* KO mice and FXS neurons from patients, which subsequently corrected the dysregulated postsynaptic organization and actin assembly, the critical processes determining synaptic maturation and density. Centrally activating autophagy in hippocampus degrades eIF4G1 and PSD-95, restores actin dynamics, and improves cognition of *Fmr1* KO mice. In human neurons derived from patients diagnosed with both FXS and intellectual disability, activating autophagy corrected the aberrant actin assembly. Thus, our findings revealed a previously unappreciated mechanism through which autophagy affects actin assembly and synaptic organization, suggesting a critical role of autophagy in regulating structural synaptic plasticity in healthy and diseased conditions.

## Introduction

Fragile X syndrome (FXS) is the most frequent form of heritable intellectual disability and the leading genetic cause of autism [1–3]. In patients with Fragile X syndrome, a CGG trinucleotide repeat located in the 5′-UTR of the fragile X messenger ribonucleoprotein 1 (*Fmr1*) gene expands from ~50 to >200, resulting in hypermethylation of the promoter region and epigenetic silencing of the *Fmr1* gene [4–7]. Fragile X Messenger Ribonucleoprotein 1 (FMRP), the gene expression product of *Fmr1*, is an RNA binding protein that tightly regulates the trafficking, localization, and translation of a vast number of neuronal mRNAs critical to neural development, synaptic plasticity, and dendritic spine architecture [1, 8–12]. Loss of FMRP and subsequent overabundance of neuronal proteins in the brains of patients and mouse models of FXS induces a complex and debilitating neurological phenotype, including impaired cognition and social interactions, hyperactivity, attentional deficits, seizures, hypersensitivity, autistic behaviors, and autonomic dysfunction [1, 2, 13–17]. However, effective treatment for FXS in humans remains unmet.

Dendritic spines are the postsynaptic compartments that receive most of the excitatory input in the brain [18]. The neuroanatomical hallmark of Fragile X is an overabundance of long and thin (immature) dendritic spines [19–22], which is associated with dysregulated group 1 mGluR-dependent long-term depression (LTD) in hippocampal neurons [23–28]. Correcting the aberrant spines has been shown to rescue, at least partially, the deficits of cognition, social behaviors, behavioral flexibility, and sensory processing in mouse models of FXS [22–29]. Importantly, recent findings reported that the altered actin dynamics critically account for the aberrant spines and related symptoms in FXS [28, 30, 31]. Actin is the most abundant cytoskeletal protein in dendritic spines and exists in dynamics between two states: monomeric globular (G-actin) or polymeric filamentous actin (F-actin) [32, 33]. F-actin provides structural support for the stability and morphology of spines [32, 33]. Dendritic spine morphogenesis, neurite formation, synapse formation/elimination, and synaptic plasticity all require fine-tuned remodeling of the actin cytoskeleton through the upstream signaling pathways [32–35]. Mouse models of FXS indicated an abnormally increased level of F-actin in the spines at cortex and hippocampus, which is causally related to increased spine density, immature spine morphology, and behavioral deficits [24, 30, 31]. To develop translational therapeutic strategies for FXS, mechanisms underlying the dysregulated spine density and morphology need to be explored.

Autophagy is a key regulator of cell growth, differentiation, and survival [36–38]. In neurons, autophagy plays an important role in protein degradation, synapse elimination, axonal homeostasis, and synaptic plasticity [23, 39–47]. At presynaptic sites, autophagy is critical to vesicular release, and impaired autophagy results in increased size of the presynaptic compartment and enhanced neurotransmitter release [48]. On the postsynaptic side, autophagy is critical to spine elimination and synapse maturation [23, 49, 50]. Cargo adaptor molecules such as p62, which bind components of the autophagic machinery, recognize and bind ubiquitinated proteins, enabling their engulfment by autophagosomes targeted for degradation [36]. Reduced autophagy in the brains of humans diagnosed with autism is associated with an accumulation of ubiquitinated proteins [49]. Recent findings revealed that autophagy is downregulated in neurons at hippocampus of a FXS mouse model [23] and in neurons derived from FXS patients [51]. Activation of autophagy leads to rescued synaptic morphology and behavioral deficits [23, 51]. However, it is still unclear how dysregulated autophagy could affect synapses and behaviors.

A critical mechanism implicated in the defects of spine morphology, exaggerated mGluR-LTD, and impaired cognition associated with Fragile X is the overabundance of neuronal proteins [1, 8, 9]. In this study, our initial analysis with proteomics revealed that 289 of the 549 overabundant proteins in the hippocampus of *Fmr1* KO mice are targets of autophagic protein degradation, indicating a strong correlation between autophagy and pathology of FXS. Further analysis indicates that these 289 proteins may mediate the correlation by affecting postsynaptic organization. Activation of autophagy rescued the aberrant postsynaptic morphology and cognitive behaviors. Proteomics analysis further narrowed that 42 of the 289 proteins may mediate the rescuing effects. Mechanistic studies revealed that among the protein targets, eukaryotic initiation factor 4G1 (eIF4G1) and postsynaptic density protein-95 (PSD-95) are critical. Autophagy degrades eIF4G1 and PSD-95 proteins, corrects the dysregulated postsynaptic organization and actin dynamics, and rescues the spine and cognitive deficits. These findings are validated with FXS mouse model with neuron-specific autophagy deficit, FXS mouse model with brain-specific autophagy activation, and human FXS neurons derived from of patient pluripotent stem cells. Altogether, our findings reveal a critical role of autophagy in regulating structural synaptic plasticity in healthy and diseased conditions and identify autophagy as a novel therapeutic target for Fragile X syndrome.

## Materials and methods

### Animals

FVB.129P2-Pde6b^+^ Tyr^c-ch^ *Fmr1*^tm1Cgr^/J (*Fmr1* KO) mice and FVB.129P2-Pde6b^+^ Tyr^c-ch^/AntJ (WT) mice were obtained from The Jackson Laboratory as described. Floxed *Atg7* (*Atg7*^loxp/loxp^) mice (C57BL/6J background, from Dr. Ana Maria Cuervo’s lab in Albert Einstein College of Medicine) and Syn1-Cre mice (B6.Cg-Tg(Syn1-cre)671Jxm/J, Jackson lab, #003966) were bred with FVB background *Fmr1* KO or WT mice for at least 5 generations. All mice were housed in a standard, pathogen-free animal facility with a 12/12 h light and dark cycle, and only male mice were used, because Fragile X syndrome is an X-linked disorder. Standard PCR was performed with tail tissues for genotyping [52]. Animal protocols were approved by the Institutional Animal Care and Use Committees of the Cleveland State University and Albert Einstein College of Medicine.

### Vectors and lentivirus

pLV-hSyn-RFP (Addgene, #22909) expressing RFP under control of Synapsin1 promoter was packaged into lentivirus with the third-generation system (VSVG, REV, and MDL, all from Addgene) and HEK293T cells (ATCC). Lenti viral vectors expressing shRNA for mouse *Eif4g1* gene (TRCN0000096813, Sigma) or control non-targeting shRNA (Sigma) were packaged into lentivirus with the second-generation system (psPAX2 and pMD2.G, from Addgene) and HEK293T cells (ATCC). High-titer lentiviral stocks were produced by calcium phosphate–mediated transfection of HEK293T and purified *via* ultra-centrifugation [53, 54]. Final virus titer was diluted to 1 × 10^6^ transducing units/μl.

### Cell culture, viral transduction, and immunocytochemistry

Primary hippocampal neurons were cultured from embryonic 18 (E18) mice, maintained in Neurobasal medium with B-27 supplement and GlutaMAX (Invitrogen), and used at DIV14 [30]. Lentivirus expressing hSyn-RFP or shRNAs were added to medium at DIV10. For immunocytochemistry, neurons were fixed with 4% PFA, blocked with 5% normal goat serum (Vector Laboratories), and subjected to reaction with primary antibodies, followed with Alexa Fluor 488, 555 or 647 conjugated secondary antibodies (Invitrogen). After 3 washing with PBS, neurons were mounted with the VECTASHIELD® Antifade Mounting Media (Vector Laboratories) with DAPI. DAPI staining was used to reveal all cells. At least three coverslips per group and multiple areas per coverslip selected on a random basis were used for analysis. ZEISS LSM 980 with Airyscan 2 super-resolution confocal microscope (20 X and 60 X objectives, averaged four times and taken at 0.6 µm depth intervals) was used to obtain consecutive Z section images. Labeled neurons were chosen randomly for quantification and the integrated puncta fluorescent intensity for a given neuron was quantified/normalized to the area of the cell body. All images were processed using the Image J software (NIH). To ensure the comparability between preparations, the same staining procedure were used, and all corresponding groups were included in each experiment. Laser settings of the microscope were uniform across all preparations.

### Human induced pluripotent stem cells (iPSCs) culture and neural differentiation

Human FXS iPSCs (WC005i-FX11-7) and (WC005i-FX08-23) and control iPSCs (WC008i-C603-4) were purchased from WiCell Research Institute (WI, USA) [55]. According to the providers’ protocol, the iPSCs were cultured and passaged in a culture medium including mTeSR™1 Medium (Stem Cell Technologies) in plates coated with Growth Factor Reduced Matrigel™ (Corning). Neural differentiation of iPSCs was performed according to previously published methods [56, 57]. Briefly, iPSCs were dissociated with TrypLE Express (Thermo Fisher Scientific), and plated on Matrigel (Corning)-coated plates in the MEF-conditioned medium with FGF-2 (Waisman Biomanufacturing), and ROCK inhibitor (Tocris Bioscience). When cells grew to nearly confluent, neural differentiation was induced with a medium including: DMEM/F12: Neurobasal medium (50/50%) (Thermo Fisher Scientific), 200 mM L-Glutamine (Thermo Fisher Scientific), 1% N2 supplement (Thermo Fisher Scientific), 0.5% B-27 supplement minus vitamin A (Thermo Fisher Scientific), and TGFβ/Smad inhibitors (10 µM SB431542 (Selleck) and 100 nM LDN193189 (Selleck)). Cells were then disassociated and re-plated on Matrigel-coated plates with the neural progenitor cell (NPC) medium including: Neurobasal medium (Thermo Fisher Scientific), 1% GlutaMAX (Thermo Fisher Scientific), 1% N2 supplement (Thermo Fisher Scientific), 0.5% B-27 (Thermo Fisher Scientific), 10 ng/ml FGF-2 and 10 µM ROCK inhibitor when plating. For neural differentiation, NPCs were re-plated on Matrigel-coated coverslips in a medium including: Neurobasal medium, 1% GlutaMAX, 1% N2 supplement, 1% B-27 minus Vitamin A, 200 nM ascorbic acid (Sigma), 1 µM cAMP (Sigma), 10 ng/ml BDNF (Cell Sciences), 10 ng/ml GDNF (Cell Sciences), 10 μM ROCK inhibitor, and 0.1 µM Compound E (Calbiochem) [58]. After two weeks, cells were fixed for immunostaining with antibodies for Tuj-1 (Mouse, R&D SYSTEMS), eIF4G1 (Rabbit, Cell Signaling), PSD-95 (Rabbit, Cell Signaling), p62 (Rabbit, MBL) and FMRP (Rabbit, Abcam). Images were acquired using a Nikon confocal microscope.

For analysis of CpG methylation in *Fmr1* promoter region, FXS and control iPSCs (20,000 cells for each sample) were collected. The bisulfite treatment of genomic DNA and pyrosequencing analysis of the *Fmr1* promoter region was performed by EpigenDx Inc (Hopkinton, MA) [55, 59].

### Cannulation and brain infusion

As we previously described [53, 54], using an ultra-precise mouse stereotactic frame (KOPF), a 26-gauge guide cannula (Plastics One, Inc.) was implanted into the lateral ventricle of anesthetized mice at the coordinates (Post bregma: 0.4 mm; Lateral to midline: 1 mm; Under bregma: 2 mm). Intra-lateral ventricular infusion was carried out with a 33-gauge internal cannula (Plastics One, Inc.) connected to a 10-µl Hamilton Syringe. Rilmenidine was dissolved in 1 µl artificial cerebrospinal fluid (aCSF) for injection. Injection of aCSF was used as vehicle control.

### In vivo adeno-associated virus (AAV) injection

Mice were anesthetized with 4% isoflurane and maintained in anesthesia with 1.5% isoflurane as described [53]. AAV encoding Syn-Cre-GFP (#105540, AAV9 from Addgene) was injected bi-laterally into hippocampus by means of a 10-µl Hamilton syringe with a 26-gauge needle with a stereotaxic frame (KOPF). The injection site was defined by the following coordinates: 2 mm posterior to bregma, 1.6 mm below the surface of the skull, and 1.8 mm lateral to the midline [53]. A total volume of 0.5 ul/ hemisphere at a flow rate of 0.1 µl/min were injected. The incision was closed with cyanoacrylate glue. After injection, animals were placed in a heated cage to recover.

### Quantitative RT-PCR

Total RNAs were isolated from hippocampal tissues using RNeasy® Mini Kit (Qiagen) and reverse-transcribed to cDNA using SuperScript™ First-Strand Synthesis System (Thermo Fisher Scientific). RNA concentration was measured by means of a Nanodrop (NanoDrop Technologies). Real-time qPCR was performed with SYBR™ Green PCR Master Mix (Thermo Fisher Scientific) for *Dlg4* (NM_001109752.1) and *Eif4g1* (NM_001005331), and normalized to *β-actin* (NM_007393). The primers used are: *Dlg-4, Forward:* 5″-TCCGGGAGGTGACCCATTC-3′; Reverse, 5′-TTTCCGGCGCATGACGTAG-3′; *Eif4g1: Forward:* 5″-AAGACCTCATCTCGCATCCG-3′; Reverse, 5′-TGTTCTCGGTGCTCTTCCATC-3′; *β-actin*: Forward, 5″-GGCTGTATTCCCCTCCATCG-3′; Reverse, 5′-CCAGTTGGTAACGCCATGT-3′. Reactions were performed in triplicate in a StepOnePlus real-time PCR system (Applied Biosystems).

### Golgi staining, spine morphology, immunolabeling and histology

The FD Rapid Golgi stain Kit (FD Neurotechnologies, MD, USA) was used to image spine morphology as described [23, 30]. In brief, mouse brains were collected, quickly rinsed, immersed in Golgi impregnation solution, and stored in the dark at room temperature for 2 weeks. Brains were then transferred and stored in Solution C for 72 hr, and cut into 150 mm-thick sections with a cryostat at −20 °C. Sections were transferred to microscope slides, rinsed, dehydrated, stained, and cleared. Spines on apical dendrites of hippocampal CA1 pyramidal neurons were imaged by means of a ZEISS LSM 980 with Airyscan 2 super-resolution confocal microscope with a 100 x oil immersion lens. Dendritic spine density was determined by counting the total number of spines along the apical dendrite from the soma to 130 μm distance on primary, secondary, and tertiary branches. Spines were classified as filopodial-like or mushroom-like/stubby in neurons using a categorization macro in Neurolucida software (MBF Bioscience), which excludes thin, branched, and detached spines [30, 60]. Five CA1 pyramidal neurons per mouse and eight 10-µm segments per neuron were analyzed.

Immunohistochemistry was performed on frozen brain sections from *Fmr1* KO and WT mice as described [23]. Mice were anesthetized, transcardially perfused with 4% PFA, and brains were removed, post-fixed for 24 hr, and infiltrated with 20–30% sucrose. 12 µm-thick brain sections were cut, blocked with normal goat serum (Vector Laboratories), incubated with primary antibodies, and then reacted with Alexa Fluor 488 or 555 secondary antibodies (Invitrogen). Naïve IgG of the appropriate species was used as a negative control. DAPI staining in mounting medium (Vector Laboratories) was used to reveal all cells in brain sections. Images were acquired using a ZEISS LSM 980 with Airyscan 2 super-resolution confocal microscope. For data analysis, serial brain sections across the hippocampus were made at the thickness of single cell (10 µm), and every 5 sections were represented by one section for staining and quantification. A minimum of three sections per mouse and multiple hippocampal CA1 regions per section were selected on a random basis and used for analysis [61]. Images were taken at 0.6-µm depth intervals. The integrated puncta fluorescent intensity for each given CA1 region was quantified and assessed using Image J software. The same staining procedure was used to ensure the comparability between preparations, and all corresponding groups were included in each experiment. Laser settings of the microscope were uniform across all preparations [23].

### Immunoprecipitation

Primary neurons were homogenized in ice-cold lysis buffer as described [23]. Hippocampal tissues were isolated from mice 4 h post treatment and homogenized with a glass homogenizer. Cell and tissue homogenates were incubated with an anti-ubiquitin (Mouse Santa Cruz) antibody or an anti-eIF4E (mouse, Santa Cruz) antibody, and gently shaken overnight at 4 °C. Supernatant with antibody was added to a slurry of IgG bound to agarose beads (Protein A/G, Pierce) and incubated with rocking at 25 °C for 2 h. Efficiency of IP was determined by comparing the abundance of immunoprecipitated protein in the supernatant and wash fractions.

### Synaptosome preparation

Briefly, 4 h post treatment, hippocampus of *Fmr1* KO and WT mice were removed, quickly rinsed with Milli-Q water, and homogenized in gradient buffer with protease and phosphatase inhibitors [62]. The homogenates were centrifuged at 1000 g for 10 min. The supernatant was loaded on a Percoll discontinuous gradient (3, 10, 15, and 23%) and centrifuged at 31,000 g for 6 min in a Beckman centrifuge. Synaptosome fractions were collected from the 15 to 23% interface and centrifuged again at 20,000 g for 10 min. The pellets were resuspended for Western blot. Protein concentrations of collected synaptosome fractions were measured with a BCA kit (Thermo Fisher Scientific).

### F-actin imaging

F-actin in cultured neurons was imaged by a high-affinity F-actin probe, phalloidin conjugated to Alexa Fluor 488 dye (ThermoFisher Scientific) [63]. Briefly, neurons were fixed, permeabilized and incubated with phalloidin staining solution at room temperature for 20 min. After being washed with PBS for 3 times, neurons were mounted with the VECTASHIELD® Antifade Mounting Media (Vector Laboratories) with DAPI. F-actin in neurons were imaged with a Nikon confocal microscope.

### Tissue preparation, Western blot, and antibodies

Hippocampal tissue was homogenized in RIPA lysis buffer supplemented with protease inhibitors (Thermo Fisher Scientific) and centrifuged at 12,000 g for 10 min at 4 °C to collect proteins. Neurons were lysed and centrifuged as above. Protein concentrations were measured with the BCA kit (Thermo Fisher Scientific) and Western blot were performed as described [23, 64]. Band densities were quantified using Image J (NIH).

Mouse hippocampus tissues were homogenized in lysis buffer with protease inhibitors as described in the manual (Cytoskeleton Inc). Large debris was removed by centrifugation at 12,000 g (10 min, 4 °C). The lysates were then incubated with GST-tagged PAK-PBD beads (Cytoskeleton Inc) for 2 h at 4 °C. GTP-Rac1 and associated proteins were precipitated from the lysates by the PAK-PBD beads. Finally, the beads were washed and resuspended in a SDS sample buffer for Western blot.

F/G-actin ratio in synaptosomes of hippocampus was assessed as previously described [30, 65]. Briefly, the synaptosome fractions were resuspended in a cold lysis buffer (10 mM K_2_HPO_4_, 100 mM NaF, 50 mM KCl, 2 mM MgCl_2_, 1 mM EGTA, 0.2 mM DTT, 0.5% Triton X-100, 1 mM sucrose, pH 7.0). Because F-actin is insoluble whereas G-actin is soluble in this buffer, F-actin and G-actin were separated by centrifuge at 15,000 g for 30 min. The F-actin pellet was resuspended in the lysis buffer mixed with another buffer (1.5 mM guanidine hydrochloride, 1 mM sodium acetate, 1 mM CaCl_2_, 1 mM ATP, 20 mM Tris-HCl, pH 7.5) at 1:1 and then incubated on ice for 1 h to convert F-actin into G-actin. The samples containing G-actin converted from F-actin were centrifuged again at 15,000 g for 30 min and the supernatant was collected for Western blot.

Primary antibodies used for Western blot include: rabbit anti-LC3-I/II (Novus), rabbit anti-p62 (MBL), rabbit anti-PSD-95 (Cell Signaling), mouse anti-Ubiquitin (Enzo), rabbit anti-ATG7 (Cell Signaling), rabbit anti-Cofilin1 (Cell Signaling), rabbit anti-phospho-Cofilin1-Ser3 (Cell Signaling), rabbit anti-eIF4G1 (Cell Signaling), rabbit anti-CYFIP1 (Millipore), rabbit anti-eIF4E (Cell Signaling), mouse anti-Rac1 (Millipore), mouse anti-puromycin (DSHB), rabbit anti-GAPDH (Cell Signaling) and rabbit anti-β-actin (Sigma). Antibodies for mouse anti-PSD-95 (Thermo Fisher Scientific), RFP (Thermo Fisher Scientific), rabbit anti-phospho-Cofilin1-Ser3 (Cell Signaling), rabbit anti-FMRP (Abcam), and eIF4G1 were used for immunocytochemistry with primary antibodies of chicken anti-MAP2 (Millipore) or mouse anti-Tuj-1 (R&D SYSTEMS). Primary antibodies of p62, Rabbit anti-GFP (Invitrogen) and mouse anti-NeuN (Millipore) were used for the immunochemistry of brain sections.

### SUnSET assay

Protein synthesis was assessed with sensing of translation (SUnSET) technique as previously described with modifications [66, 67]. Briefly, primary neurons were treated with 5 µg/ml puromycin (Sigma) for 30 min. Protein synthesis was examined as puromycin incorporation in new synthesized proteins of cell lysates by Western-blot using an anti-puromycin antibody (PMY-2A4, DSHB). Western-blot of GAPDH (Cell Signaling) was used as a loading control.

### Novel object recognition test

The novel object recognition task was conducted in an isolated arena (40 cm length x 40 cm width and x 40 cm height) [68]. For habituation before the testing day, mice were allowed to explore the empty arena for 10 min. On the testing day (training and familiarization session), the mouse was placed in the center of the arena between, and equidistant from, two identical objects and allowed to freely explore for 10 min. The mouse was then placed in a holding cage for 24 h. The next day (test session), one of the objects was replaced with a novel object. The mouse was placed in the arena for an additional 10 min. The time spent exploring each object was recorded by investigators blind to the grouping information with stopwatches. Mice’s movements were also recorded with ANY-maze Video Tracking System. Mice that did not spend a minimum of 10 s investigating one or both objects were excluded from the study. The preference index was calculated by dividing the time exploring the novel object by the total time exploring the two objects. Exploration was defined as orienting the nose toward the object with a distance <2 cm between the nose and the object. Resting, grooming, or sitting on the object was not considered as exploration.

### Contextual fear conditioning

Cognition test with contextual fear conditioning was performed in a Freezeframe Chamber and analyzed by Actimetrics Software (Actimetrics) as previously described [68]. On the day of fear conditioning (day 1), mice were habituated in chamber 1 for 3 min, followed by two shocks of 0.7 mA (1 s each). Mice then remained in chamber 1 for 15 s after the shock. On day 2, mice were separated into two groups: one group was tested in chamber 1 in the same context with day 1 (familiar context), and the others were tested in chamber 2 with a different (novel) context. The percentage of time that a mouse shows freezing response in the 3 min test session on day 2 was recorded by the software.

### Nest building assay

Nest building was assessed as described [25, 69]. Mice were single housed with a 2.5 g Nestlet and left undisturbed for 24 h. Nests were assessed on a rating scale of 1–5 as described before [69]. Untorn nest pieces were weighed.

### Open field and self-grooming tests

The open field test was performed in a 40 × 40 × 40 cm^3^ arena for 10 mins [68]. The floor of the arena was divided into two zones: an ‘inner’ zone (containing the inner 25 × 25 cm^2^ center square) and an ‘outer’ zone (the outermost area 15 cm from the walls). The times spent in ‘inner’ zone and ‘outer’ zone were recorded by investigators blind to the grouping information with stopwatches. Mice’s movements were recorded with ANY-maze Video Tracking System. Times spent in self-grooming were also recorded by investigators blind to the grouping information with stopwatches during the test.

### HPLC-MS/MS

The HPLC-MS/MS method was performed with a Shimadzu UPLC system (Columbia, MD), which consisted of a Prominence DGU-20A_3R_ inline degasser, two LC-30 AD pumps, a SIL-30 AC autosampler and a CBM-20A controller. The chromatographic separation was performed on a Kinetex C_18_ column (50 mm × 2.1 mm, 1.3 µm) with a mobile phase consisting of acetonitrile-0.1% formic acid and water (50:50, v/v) at a flow rate of 0.3 ml/min. The temperature of the column was maintained at 36 °C. The injection volume was 5.0 µl. Mass spectrometric detection was operated on an AB Sciex Qtrap 5500 mass spectrometer (Toronto, Canada) with negative electrospray ionization mode. The multiple reaction monitoring (MRM) function was used for quantification with the transitions of Rilmenidine and IS trimipramine-d3, which were detected at m/z 180.9 → 66.9 and m/z 297.8 → 103.2, respectively. The optimized ion source parameters were set as follows: ion spray voltage, 2000 V; ion source temperature, 550 °C; nebulization gas 40 psi; auxiliary gas, 40 psi; curtain gas, 30 psi. Compound parameters were as follows: Rilmenidine: declustering potential, 23 V; entrance potential, 6.5 V; collision energy, 28 V; Collision entrance potential, 15 V. Trimipramine-d3: declustering potential, 40 V; entrance potential, 5 V; collision energy, 25 V; Collision entrance potential, 15 V. The stock solutions were prepared by dissolving Rilmenidine and trimipramine-*d3* in methanol at 1.0 mg/ml. Then, the stock solution of Rilmenidine was serially diluted with methanol into a concentration gradient: 0.5, 1.0, 2.0, 5.0, 10, 20, 50, 100, 200, 500, 1000 ng/ml. Also, a 500 ng/ml working solution of trimipramine-*d3* (IS) was prepared in methanol from its stock solution. The calibration standards were prepared as follows: after spiking with 100 µl of the corresponding standards solutions, 40 µl of trimipramine-*d3* working solution, 100 µl of blank mouse plasma or brain homogenates (0.4 g blank brain tissue mixed with 2 ml PBS), and 800 µl of methanol were transferred into a 1.5 ml tube, and the mixture was then vortexed and centrifuged at 12,000 g for 10 min. The supernatant was collected and dried with nitrogen, and then the residue was stored at −80 °C and dissolved with 50% acetonitrile before analysis. A protein precipitation method was applied to extract Rilmenidine from mouse plasma and brain homogenate (0.4 g brain tissue mix with 2 ml PBS). Briefly, 100 µl of each sample, 40 µl of trimipramine-*d3* (IS, 500 ng/ml), and 800 µl of methanol were combined in a 1.5 ml tube. Then, it was vortexed and centrifuged, and the supernatant was collected and dried with nitrogen as the calibration standard. The residue was stored at −80 °C and dissolved with 100 µl 50% acetonitrile before analysis.

### Proteomics

Hippocampus were collected from mice 4 h post treatments., homogenized, and analyzed with a tandem mass tags (TMT) labeling technique by Proteomics & Metabolomics Core, Lerner Research Institute, Cleveland Clinic [70]. Briefly, each of the mouse hippocampus was suspended in 150 μl 8 M urea Tris-HCl pH8 lysis buffer with freshly added protease inhibitor cocktail. Samples were homogenized by ultrasonication 15 s x 3 with 15 s intervals. Homogenized samples were centrifuged at 15000 g for 15 min, and the supernatants were transferred to new 1.5 ml tubes. Protein concentrations of the samples were determined by a BCA kit. 50 μg of protein from each sample were taken. The samples were reduced by dithiothreitol, alkylated by iodoacetamide, and precipitated by cold acetone (−20 °C) overnight. Samples were centrifuged at 8000 g for 5 min at 4 °C, and the supernatants were removed. Protein pellets were air-dried, dissolved, digested overnight. Digested peptide samples were labeled with TMTpro 16plex tags according to the protocol from the manufacturer’s instruction. The Thermo Scientific Fusion Lumos mass spectrometry system with the Dionex 15 cm × 75 µm id Acclaim Pepmap C18, 2μm, 100 Å reversed-phase capillary chromatography column was used. 5 μl volumes of the extract were injected and the peptides eluted from the column by an acetonitrile/0.1% formic acid gradient at a flow rate of 0.3 μl/min were introduced into the source of the mass spectrometer on-line. The digest was analyzed using a TMT-MS2 method. Over 4900 proteins were identified in the samples. The results of proteomics were first subjected to overlap analysis. To assess the significance of the overlap between the protein lists, hypergeometric tests were performed. The hypergeometric distribution models show the probability of the number of overlapping genes between two subsets drawn without replacement. The null hypothesis posits that the overlap between each two lists is due to random chance. The cumulative distribution function (CDF) of the hypergeometric distribution was used to compute the probability of observing an overlap greater than or equal to the observed value. The lists of overlapped proteins were then analyzed by PANTHER Overrepresentation Test (PANTHER 18.0) with *Mus Musculus* database as a reference list. The protein candidates were classified into annotated GO categories of biological processes/cellular components and compared with the *Mus Musculus* database of brain-expressed genes (https://mouse.brain-map.org/) as the background to determine whether they are overrepresented or underrepresented for a given GO biological process/cellular component. For the SynGO ontology enrichment analyses, we uploaded 289 identified proteins (Gene ID) to the website (https://www.syngoportal.org/index.html) and compared with database of synaptic proteins (updated version 20231201). Fold enrichment is defined as the ratio of proteins classified in each GO category from the experimental dataset relative to the number of proteins predicted to be in the same GO category from the reference dataset. Bonferroni correction for multiple testing was applied for statistics.

### Statistical analysis

Statistical analysis used is detailed in figure legends. Data are presented as the mean ± s.e.m. The expected sample sizes for cell cultures and animal studies were estimated based on analysis with G*Power 3.1 software and our previous studies. Mice that met the inclusion criteria (described in legends) were randomly assigned to experimental and control groups using a computer-generated random number sequence to ensure unbiased allocation. To minimize bias, researchers conducting tests and data collection were blinded to group allocation. Neurons were allocated to treatment or control groups using a random number. All plates and wells were labeled with anonymous codes, and experimenters were blinded to the treatment conditions until data analysis. The Kolmogorov–Smirnov test was used to analyze normal distribution of data. The student’s t-test (unpaired) and one-way ANOVA with post hoc Tukey’s test, was used to establish statistical significance using Originpro (OriginLab). All tests are two-sided when applicable. The variance between groups was assessed with Levene’s test (*p* < 0.05) using Originpro (OriginLab), which indicated no significant differences in variance. *n* = the number of animals or biological repeats (cultures) used in the analysis. For animal experiments, animals exhibiting signs of illness or significant deviations in weight (>10% deviation from group mean) were excluded. Cell samples were excluded if they met any of the following criteria: Low viability (<90%), Microbial contamination, Morphological abnormalities. All the exclusion criteria were pre-established. Statistical significance was defined as *p* < 0.05. Bonferroni correction for multiple testing was applied. Specific sample numbers, including the numbers of cell culture, repeats or mice, are indicated in the figure legends.

## Results

### Proteomic analysis reveals the correlation between downregulated autophagy and FXS

**Fig. 1:**
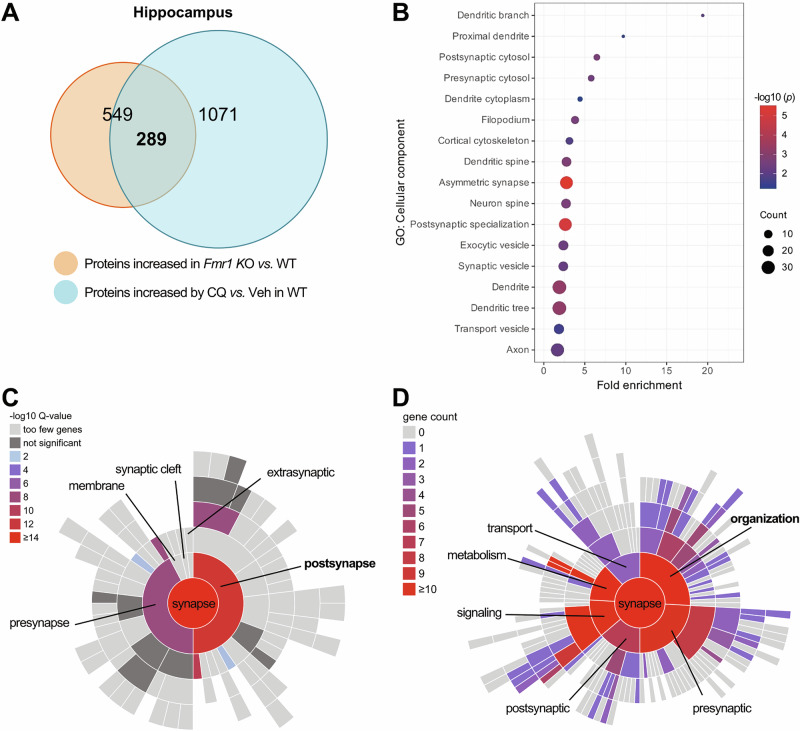
Proteomic analysis on hippocampus of *Fmr1* KO mice and mice with inhibited autophagy. Hippocampal tissues were isolated from *Fmr1* KO (*Fmr1* KO) *vs*. wild-type (WT) mice (5-week-old), and WT mice (5-week-old) injected (*i.p.)* with saline as vehicle (Veh) or Chloroquine (CQ, 50 mg/kg BW). Total protein lysates were analyzed by proteomics. **A** Venn diagram showing 289 overlapped proteins between proteins significantly increased (*p* < 0.05) in *Fmr1* KO *vs*. WT mice and proteins significantly increased (*p* < 0.05) in mice injected with CQ *vs*. Veh. The significance of overlap: *p* < 10^−16^. **B** GO “cellular component” analysis of 289 overlapped proteins (Mus musculus database of brain-expressed genes as the background, Dataset S4; and top neuron-specific components were shown. Color intensity depicts −log10(*p* value) and the size of circle denotes the number of proteins associated with each component. **C** Sunburst blot showing the SynGO [74] locations and enrichment in each term on a color-coded scale as indicated. The blot is organized from the parent term, “synapse” in the center, to successively more refined child terms in the outer shells. **D** Sunburst blot showing the SynGO biological processes and the number of proteins in each process on a color-coded scale as indicated. The blot is organized from the parent term, “synapse” in the center, to successively more refined child terms in the outer shells. n = 4 mice in each group.

FXS individuals and animal models are characterized with overabundance of hundreds of neuronal proteins [1, 8, 9]. Although most of the overabundances are moderate, the affected proteins together crucially induce complex dysregulated signal pathways, aberrant synapses, and behavioral deficits in FXS [1, 8, 9]. To maintain the correct number of proteins, the balance between protein synthesis and degradation must be fine‐tuned. In neurons, autophagy plays an important role in protein degradation [39]. Brains from humans diagnosed with autism show reduced autophagy and accumulated ubiquitinated proteins [49]. We previously reported that autophagy is downregulated in hippocampal neurons of *Fmr1* KO mice [23]. To estimate the role of downregulated autophagic protein degradation in synaptic defects of FXS, we compared proteins increased in hippocampus of *Fmr1* KO (*Fmr1*^-/y^) mice *vs*. WT mice with proteins increased in hippocampus of WT mice when autophagy was inhibited by a pharmacological inhibitor, Chloroquine (CQ) [71]. The results indicated that 549 proteins were significantly increased (*p* < 0.05) in hippocampus of *Fmr1* KO mice *vs*. WT mice (Fig. 1A and Dataset S1). 289 of these 549 proteins (52.6%) overlapped with proteins significantly increased in hippocampus of mice injected with CQ *vs*. Vehicle (Dataset S2), indicating that these 289 proteins are either directly degraded by autophagy or indirectly affected by the downregulated autophagy. Thus, the result indicated that downregulated autophagy plays an important role in the protein overabundance and pathology of FXS.

To further estimate the contribution of autophagy to FXS, we performed a Gene Ontology (GO) biological processes enrichment analysis based on the 289 proteins. Fragile X syndrome is a neurodevelopmental disorder, and its pathology primarily affects synaptic functions and morphology in neurons [2, 48]. The GO analysis results indicated that “Synaptic vesicle priming”, “Dendritic spine development”, “Dendrite development”, “Regulation of synapse structure or activity”, and “Synapse organization” are among the most statistically enriched GO terms (Supplemental Fig. 1 and Dataset S3), suggesting that these 289 proteins are tightly related to the morphology and functions of synapses and dendritic spines, where most of the post-synaptic sites located. Proteins need to be located at certain cellular components to execute the relevant biological processes. Thus, profiling the subcellular components where proteins are located can further explore their roles [72]. GO analysis on Cellular Component (GOCC) with background of brain expression genes demonstrated that synapses and spines are the top (Fold enrichment >1 and *p* < 0.05) neuronal subcellular components where these 289 proteins are located (Fig. 1B and Dataset S4). The aberrant spines, synapses and neural circuits are considered as neurological basis for cognitive and behavioral deficits in FXS [48, 73]. Indeed, the Synaptic Gene Ontologies (SynGO) database (version 20231201) [74] shows the most enriched synaptic component for these 289 proteins is the postsynaptic site (Fig. 1C and Dataset S5). SynGO biological process analysis further indicated that the biggest number of the genes are involved in the process of “organization’ of synapses (Fig. 1D and Dataset S6). Thus, our findings suggest that impaired autophagy crucially contributes to the pathology of FXS, and the protein targets affecting postsynaptic organization in hippocampus may mediate the process. Restoring the impaired autophagy may potentially rescue synaptic and cognitive deficits.

### Pharmacological activation of autophagy in hippocampal neurons of *Fmr1* KO mice

**Fig. 2:**
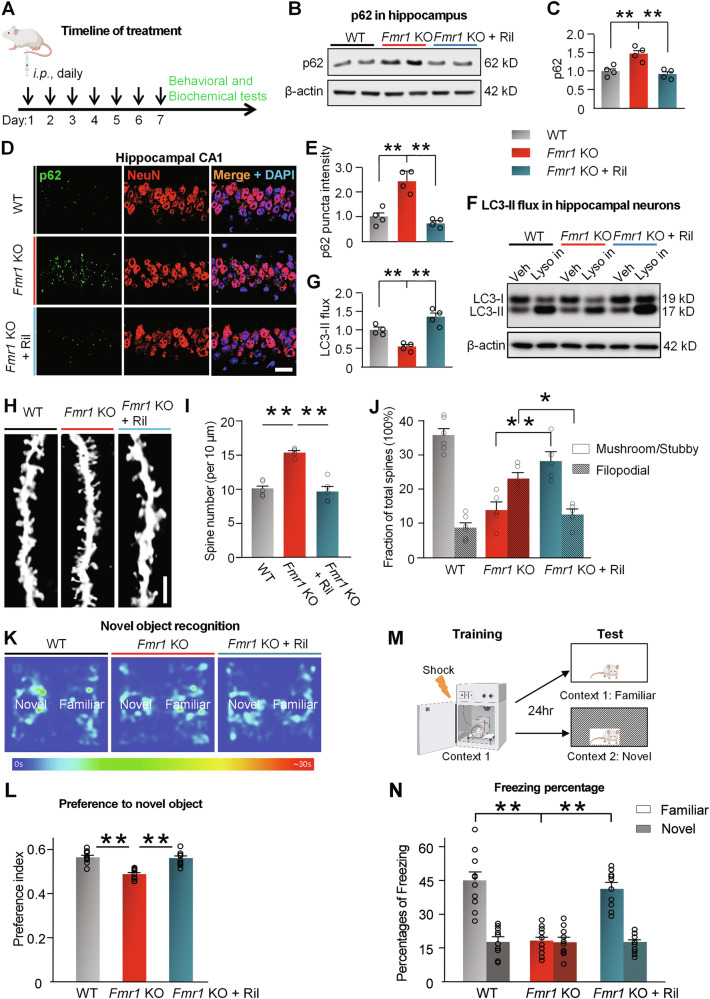
Activation of autophagy rescued the synaptic and cognitive deficits of *Fmr1* KO mice. **A** Schematic for Rilmenidine treatment: Wild-type (WT) mice were injected daily with vehicle (saline) and *Fmr1* KO mice were injected daily with vehicle (*Fmr1* KO) or Rilmenidine (*i.p*., 10 mg/kg BW) (*Fmr1* KO + Ril) for 1 week. **B** Hippocampal lysates from treated mice were assessed with Western blot for p62. **C** Bar graph shows summary of normalized data of B. **D** Brain frozen sections were subjected to immunostaining of p62 together with NeuN to mark neurons. Scale bar, 25 µm. **E** Summary bar graph shows the normalized fluorescent intensity of p62 puncta of D. **F** Primary neurons were cultured from the hippocampus of WT and *Fmr1* KO mice and treated with Veh (DMSO) or Rilmenidine (10 µM for 6 hr). Neurons from each group were treated with/without lysosomal inhibitors (Lys Inh, 20 mM NH_4_Cl and 100 μM leupeptin) in the last 2 h. Protein lysates were assessed with Western blot of LC3. **G** Bar graph shows summary data of F. LC3-II flux is quantified by subtracting LC3-II densitometric value of samples without Lyso Inh from corresponding lysosomal inhibitors-treated samples. **H** Brains of treated mice were subjected to Golgi staining and all spines located on apical dendrites on CA1 pyramidal neurons were analyzed. Scale bar, 5 µm. **I** Spine number per 10 μm of dendrite. (5 mice, 25 neuron, and 2020 spines in WT; 5 mice, 25 neuron, and 3100 spines in *Fmr1* KO; 5 mice, 25 neuron, and 1900 spines in in *Fmr1* KO + Ril were analyzed). **J** Analysis of stubby/mushroom and filopodial spine fractions. (6 mice, 30 neuron, and 2424 spines in WT; 5 mice, 25 neuron, and 3100 spines in *Fmr1* KO; 5 mice, 25 neuron, and 1900 spines in in *Fmr1* KO + Ril were analyzed). **K, L** Visual memory was assessed by the novel object recognition task: **K** Representative heatmaps of mouse movement; **L** Preference index to novel object (n = 9 mice in WT, n = 10 in *Fmr1* KO, and n = 10 in *Fmr1* KO + Ril). **M** Design of contextual fear condition test. (*Created in BioRender. Yan, J. (2025)*) **N** Percentages of freezing response in familiar and novel contexts during the test session (n = 10 mice in each group). Significance was calculated by the t-test (unpaired, two-tailed) and one-way ANOVA followed by a Tukey’s test. *p < 0.05. **p < 0.01. β-actin was used as a loading control. Values reflect mean ± s.e.m. Each circle represents data from an individual mouse (in C, E, I, J, L and N) or an independent culture (in G, n = 4 cultures). n = 4 mice in C and E. Mice are 5-week-old.

Rilmenidine is an FDA-approved, blood-brain barrier (BBB) permeable anti-hypertensive agent by activating ADRA2/a2-adrenoceptors, imidazoline-1 receptors and sympathetic nervous system in the brain [75, 76]. In addition, Rilmenidine also activates autophagy, improves energy metabolism, reduces oxidative stress, and affects ageing processes [75–78]. Rilmenidine can significantly activate autophagy in neurons of a Huntington’s Disease mouse model and a mutant SOD1-induced amyotrophic lateral sclerosis mouse model [79, 80]. Pharmacokinetic assay with HPLC-MS/MS shows after intraperitoneal (*i.p*.) injection, Rilmenidine crossed the BBB, leading to comparable concentrations in the brain and plasma (Supplemental Fig. 2A, B). To optimize the dose and timeline for Rilmenidine injection, we tested the dose and time effects of Rilmenidine on autophagy in mouse hippocampus. When autophagy flux is inhibited, the cargo adaptor protein, p62 accumulates [36–38]. Two hours post injection, both 10 mg/kg and 100 mg/kg dosages significantly reduced p62 protein levels, indicating activated autophagy in the hippocampus (Supplemental Fig. 3A). Time-course effect indicated that 10 mg/kg of Rilmenidine injection started to significantly reduce p62 levels 2 h post injection, and the effect lasted until 8 h post injection (Supplemental Fig. 3B). Thus, to activate autophagy in brain, mice received daily *i.p*. injection of Rilmenidine at 10 mg/kg for 1 week [79] (Fig. 2A). This treatment did not significantly affect the body weight and length of mice (Supplemental Fig. 4).

Consistent with the proteomics data, p62 was markedly increased in the hippocampus from *Fmr1* KO *vs*. WT mice (Fig. 2B, C). Rilmenidine reduced the elevated p62 abundance in *Fmr1* KO mice to a similar level as WT mice. Immunostaining of p62 with brain sections shows that Rilmenidine reduced the accumulated p62 proteins in CA1 neurons of *Fmr1* KO mice (Fig. 2D, E). Upon initiation of autophagy, LC3-II becomes associated with the autophagosomal membrane and is subsequently degraded in lysosomes as a part of autophagic cargo [36]. We next assessed autophagy flux as rates of LC3-II turnover by comparing LC3-II levels in the presence and absence of lysosomal inhibitors [81]. Net LC3-II flux decreased in hippocampal neurons cultured from *Fmr1* KO mice *vs*. WT mice, which was reversed by treating with Rilmenidine (Fig. 2F, G). Because neurons lack ability to dilute damaged material through cell division [82], efficient and quick autophagic degradation of cargos are required to maintain the neuronal health [82, 83]. In hippocampal neurons of wild type mice, where autophagy is already efficient in normal condition, Rilmenidine treatment only slightly reduced cargo protein p62 levels (Supplemental Fig. 5A–D). Collectively, these findings indicate that Rilmenidine restores the downregulated autophagy in the hippocampus of *Fmr1* KO mice.

### Activation of autophagy mitigated the aberrant spine and cognitive deficits in *Fmr1* KO mice

Hippocampal neurons of patients with FXS and *Fmr1* KO mice exhibit an excess of dendritic spines and immature spine morphology [27, 60]. To examine the effect of pharmacological activation of autophagy on spine morphology, we first injected *Fmr1* KO mice and WT mice with Rilmenidine or vehicle as seen in Fig. 2A and assessed dendritic spine morphology (Fig. 2H). *Fmr1* KO mice showed increased spine density on dendrites of CA1 pyramidal neurons compared with WT mice, while Rilmenidine corrected the increased spine density of *Fmr1* KO mice to near that of WT mice (Fig. 2I). Next, we examined the impact of Rilmenidine on spine maturation by classifying spines as stubby and mushroom-shaped (mature) or spindly, filopodial-like protrusions (immature). CA1 neurons from *Fmr1* KO mice exhibited a marked decrease in the percentage of mushroom/stubby spines and a marked increase in the percentage of long, filopodial-like protrusions, relative to that of WT (Fig. 2J), consistent with previous findings [23, 27, 60]. Rilmenidine increased the percentage of mature spines of *Fmr1* KO mice to near WT levels (Fig. 2J). Rilmenidine treatment did not significantly alter the spine density in WT hippocampal CA1 neurons and only slightly reduced percentage of immature spines (Supplemental Fig. 5E–G). Thus, activation of autophagy by Rilmenidine corrected abnormalities in the spine density/morphology of hippocampal neurons in *Fmr1* KO mice.

Patients with FXS exhibit cognitive deficits [1, 2, 84] and *Fmr1* KO mice display deficits in visual memory [85]. We next examined the impact of activating autophagy on cognition of *Fmr1* KO mice. The novel object recognition task assesses visual memory and takes advantage of the innate tendency of wild-type mice to spend more time exploring a novel *vs*. a familiar object [68]. Vehicle-treated WT mice showed a strong preference for the novel object (Fig. 2K, L and Supplemental Fig. 6). *Fmr1* KO mice injected with vehicle spent approximately equal times exploring the novel and familiar objects, indicating no preference for the novel object and impaired cognition. *Fmr1* KO mice injected with Rilmenidine spent more time exploring the novel *vs*. familiar object, indicating preference to novel object and rescued cognition (Fig. 2K, L and Supplemental Fig. 6). We next examined the effects of Rilmenidine on contextual memory with the contextual fear condition test. In this assay, mice were exposed to a distinctive environmental context in which they received a shock. On the testing day (24 h after the shock), they were returned to either the same (familiar) or a different (novel) context (Fig. 2M). *Fmr1* KO mice exhibited profound memory deficits on the testing day, as evidenced by lack of freezing response in the familiar context (Fig. 2N). *Fmr1* KO mice treated with Rilmenidine indicated a significantly higher percentage of freezing in the familiar context on testing day, comparable to *Fmr1* KO mice treated with the vehicle, demonstrating that activating autophagy enhanced the memory of the association between context and an aversive event. In addition to cognitive tests, effects of Rilmenidine on other behavioral deficits reported with *Fmr1* KO mice were also examined [25, 86]. Rilmenidine treatment failed to rescue the impaired nest building behavior (Supplemental Fig. 7A, B) and the increased center time (open field test, Supplemental Fig. 7C, D) of *Fmr1* KO mice. *Fmr1* KO mice exhibited higher levels of self-grooming, a repetitive behavior [86], which are significantly reduced by Rilmenidine (Supplemental Fig. 7E). Collectively, Rilmenidine corrected abnormalities in spine density/structure and cognitive deficits associated with *Fmr1* KO mice.

## The rescuing effects on FXS phenotypes rely on activation of autophagy in neurons

To identify the role of dysfunctional autophagy in cognitive deficits in FXS, we first knocked down *Atg*7 (autophagy-related 7 gene), a key component of autophagy, in hippocampal neurons of wild type mice and then observed their cognitive behaviors. Adino-associated virus (AAV) expressing Syn-Cre-GFP were injected bilaterally to hippocampus of wild type (*Atg7*^w/w^), heterozygous *Atg7* floxed (f) mice (*Atg7*^w/f^), and homozygous *Atg7*^f/f^ mice (Supplemental Fig. 8A–C). The expression of Cre significantly reduced ATG7 protein level and increased p62 levels in hippocampus of both heterozygous *Atg7*^w/f^ and homozygous *Atg7*^f/f^ mice, indicating compromised autophagy (Supplemental Fig. 8D–F). Hippocampal neuronal knockdown of *Atg*7 induced deficits in the visual memory of *Fmr1* KO mice, demonstrated by the decreased preference to the novel object (Supplemental Fig. 8G–I). Expression of Cre in hippocampal neurons also lead to decreased freezing response in both heterozygous *Atg7*^w/f^ and homozygous *Atg7*^f/f^ mice, indicating cognitive deficits (Supplemental Fig. 8J). Thus, knockdown of *Atg*7 in hippocampal neurons leads to similar cognitive deficits as observed in FXS, suggesting that dysfunctional autophagy in hippocampal neurons plays a critical role in this process.

**Fig. 3:**
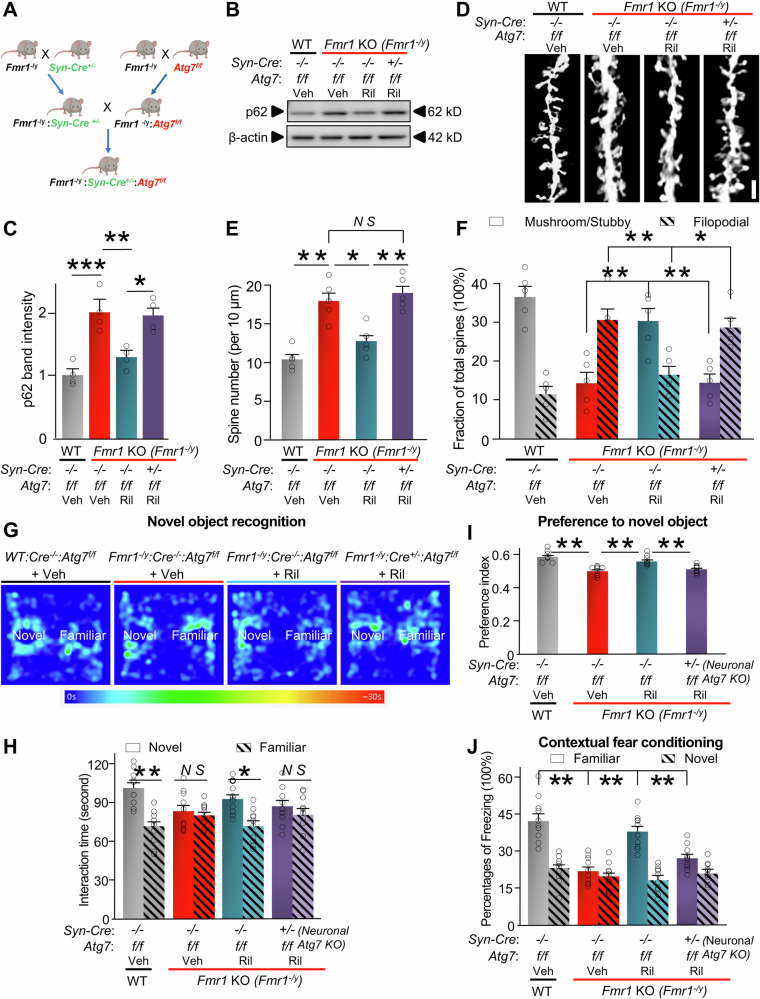
Neuronal specific *Atg7* knockout attenuated the rescue effects of Rilmenidine. **A** Schematic showing breeding of *Fmr1* KO (*Fmr1*^*-/y*^) mice with neuron-specific *Atg7* knockout (*Fmr1*^*-/y*^: Cre^+/−^: *Atg7*^f/f^ mice). **B** *Fmr1*^*-/y*^: Cre^+/−^: *Atg7*^f/f^ mice*, Fmr1*^-/y^: Cre^−/−^: *Atg7*^f/f^ mice (*Fmr1* KO control), and WT: Cre^−/−^: *Atg7*^f/f^ mice (WT control) were injected (*i.p*.) with vehicle (Veh) or Rilmenidine (Ril) daily for one week and lysates of hippocampus were assessed with Western blot for p62. β-actin was used as a loading control. **C** Bar graph shows normalized summary data of B (n = 4 mice in each group). **D** Brains were isolated and subjected to Golgi staining. Spines located on apical dendrites on CA1 pyramidal neurons were analyzed. Scale bar, 3 µm. **E** Spine numbers per 10 μm of dendrite. **F** Analysis of stubby/mushroom and filopodial spine fraction. (For E and F, 5 mice, 25 neuron, and 2092 spines in WT: Cre^−/−^: *Atg7*^f/f^ mice; 5 mice, 25 neuron, and 3580 spines in *Fmr1*^-/y^: Cre^−/−^: *Atg7*^f/f^ mice; 5 mice, 25 neuron, and 2540 spines in *Fmr1*^-/y^: Cre^−/−^: *Atg7*^f/f^ mice + Ril; 5 mice, 25 neuron, and 3780 spines in *Fmr1*^-/y^: Cre^+/−^: *Atg7*^f/f^ + Ril mice were analyzed.) **G-I** Visual memory was assessed by means of the novel object recognition task: **G** Representative heatmaps of mouse movement. **H** Time spent exploring novel and familiar objects (n = 10 mice in each group). **I** Preference index to novel object. **J** Percentages of freezing response in familiar and novel contexts during the contextual fear conditioning task (n = 10 mice in each group). Significance was calculated by the t-test (unpaired, two-tailed) and one-way ANOVA followed by a Tukey’s test. *p < 0.05. **p < 0.01. ***p < 0.001. *N S*: no significant difference. Values reflect mean ± s.e.m. Each circle represents data from an individual mouse. Mice used are 5-week-old.

Rilmenidine activates autophagy and affects several cellular processes, downstream of imidazoline receptors [75, 87]. To distinguish the contribution of neuronal autophagy to the rescuing effect of Rilmenidine, we next examined whether neuron-specific knockdown of *Atg7* can reverse the drug-induced rescue. To do so, we bred WT or *Fmr1* KO (*Fmr1*^-/y^) mice with *Atg7*^f/f^ mice and Synapsin1-Cre mice (*Cre*^*+/*−^), resulting in WT and *Fmr1* KO mice with neuron-specific *Atg7* knockout (Fig. 3A). From these crosses, we obtained the compound mice, termed WT: *Cre*^−/−^: *Atg7*^f/f^ (as WT control), *Fmr1*^-/y^: *Cre*^−/−^: *Atg7*^f/f^ (as *Fmr1* KO control), and *Fmr1*^-/y^: *Cre*^+/−^: *Atg7*^f/f^ (*Fmr1* KO mice with neuron-specific *Atg7* knockout). We then injected Rilmenidine or vehicle into these mice as in Fig. 2A. *Atg7* knockout was confirmed with reduced ATG7 protein expression in hippocampal tissues of the *Fmr1*^-/y^: *Cre*^+/−^: *Atg7*^f/f^ mice (Supplemental Fig. 9). Then, we examined autophagy activity in the hippocampus. *Fmr1* KO control mice (*Fmr1*^-/y^: *Cre*^−/−^: *Atg7*^f/f^) injected with the vehicle showed increased p62 levels in hippocampal tissues *vs*. WT controls (WT: *Cre*^−/−^: *Atg7*^f/f^) injected with the vehicle (Fig. 3B, C). Rilmenidine significantly reduced p62 protein levels in *Fmr1* KO control (*Fmr1*^-/y^: *Cre*^−/−^: *Atg7*^f/f^) mice but failed to do so when *Atg7* is neuron-specifically knocked out (*Fmr1*^-/y^: *Cre*^+/−^: *Atg7*^f/f^). Rilmenidine successfully corrected the increased spine density of *Fmr1* KO mice but failed to do so in the *Fmr1* KO mice with neuron-specific *Atg7* knockout (Fig. 3D, E). Moreover, Rilmenidine failed to increase the percentage of mature spines in the *Fmr1* KO mice with neuron-specific *Atg7* knockout (Fig. 3D, F). Behavioral tests indicated that Rilmenidine administration significantly improved cognition as measured by novel object recognition (Fig. 3G, H, I) and contextual fear conditioning (Fig. 3J) in *Fmr1* KO control mice but not in *Fmr1* KO mice with neuron-specific *Atg7* knockout. Thus, inhibition of autophagy in neurons largely compromised the rescue effects, indicating that the rescuing relies on activation of autophagy in neurons.

## Identification of downstream protein targets mediating autophagy’s rescuing effects

**Fig. 4:**
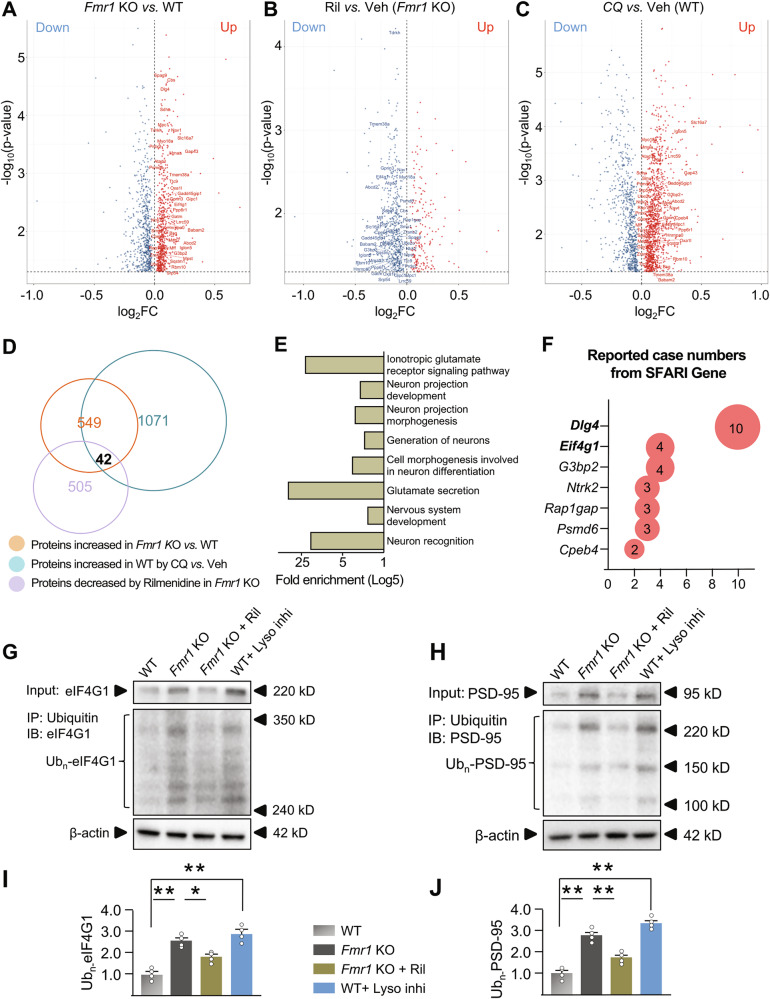
Identifying the protein targets bridging autophagy to aberrant spines. Hippocampus were isolated from *Fmr1* KO *vs*. WT mice with different treatments and protein lysis was subjected to proteomics. **A** Volcano plot for proteins significantly (*p* < 0.05) up- (red) or down-regulated (blue) in *Fmr1* KO *vs*. WT mice. **B** Volcano plot for proteins significantly (*p* < 0.05) up- (red) or down-regulated (blue) in *Fmr1* KO mice injected with Rilmenidine (Ril) *vs*. saline (Veh). **C** Volcano plot for proteins significantly (*p* < 0.05) up- (red) or down-regulated (blue) in WT mice injected with CQ (autophagy inhibitor) *vs*. Veh. **D** Venn diagram showing overlapped 42 proteins. Protein names are labeled in A, B, and C. Overlap between proteins increased in *Fmr1* KO vs. WT and proteins increased in WT by CQ *vs*. Veh: *p* < 10^−16^. Overlap between proteins decreased by Rilmenidine in *Fmr1* KO and proteins increased in WT by CQ *vs*. Veh: *p* = 1.4 × 10^−9^. Overlap between proteins increased in *Fmr1* KO vs. WT and proteins decreased by Rilmenidine in *Fmr1* KO: *p* < 10^−16^. **E** GO biological processes analysis (*Mus musculus* database of brain-expressed genes as the background, Dataset S8) of 42 overlapped proteins showing top nervous system-related processes (*p* < 0.05). **F** SFARI Gene database shows 7 of these 42 overlapped proteins are associated with reported autism cases. **G,** **H** Primary neurons were cultured from the hippocampus of WT and *Fmr1* KO mice and treated with labeled drugs: WT (WT neurons with DMSO); *Fmr1* KO (*Fmr1* KO neurons with DMSO); *Fmr1* KO + Ril (Rilmenidine, 10 µM for 6 hr) and WT + lyso Inhi (lysosomal inhibitors: 10 mM NH_4_Cl with 50 μM leupeptin for 6 hr). Lysates were extracted and immunoprecipitated with an antibody to ubiquitin. Whole cell lysates (Input) and immunoprecipitants (IP) were immunoblotted (IB) for eIF4G1 and PSD-95. **I, J** Summary data for ubiquitinated eIF4G1 and PSD-95 in precipitates (normalized to WT + DMSO). n = 4 mice (5-week-old) in each group of A-C. Significance were calculated by ANOVA followed by a Tukey’s test. *p < 0.05, **p < 0.01. β-actin was used as a loading control. Each circle in I and J represents data from an independent culture (n = 4).

To investigate the mechanisms underlying how activated autophagy regulates spine density/morphology, we conducted proteomics to profile the altered proteins in hippocampus of *Fmr1* KO mice injected with Rilmenidine. Indeed, we identified 549 proteins that significantly increased in the hippocampus of *Fmr1* KO mice *vs*. WT mice (Fig. 4A). Importantly, 42 of these 549 proteins were successfully reduced by Rilmenidine in *Fmr1* KO mice (Fig. 4B), and identified as direct or indirect targets of autophagic protein degradation as being significantly upregulated by the autophagy inhibitor in WT mice (Fig. 4C). Thus, these overlapped 42 proteins (Fig. 4D, Dataset S7, and labeled in Fig. 4A–C) may serve as the downstream targets of autophagy to rescue the synaptic and cognitive deficits of FXS. GO biological process analysis confirmed that these 42 proteins play important roles in neurons and synapses. “Glutamate secretion”, “Ionotropic glutamate receptor signaling pathway”, “Neuron recognition”, “Cell morphogenesis involved in neuron differentiation” and “Neuron projection morphogenesis” are among the most significant categories (Fig. 4E, Dataset S8). To further identify the relevance of these 42 proteins with autism-related synaptic and behavioral deficits, we searched the SFARI Gene database. Searching results revealed that mutations of 7 among these 42 genes (*Dlg4, Eif4g1, G3bp2, Ntrk2, Rap1gap, Psmd6 and Cpeb4*, as shown in Fig. 4F) are associated with autism cases and *Dlg4* (encoding PSD-95 protein) and *Eif4g1* (encoding eIF4G1 protein) are reported with the highest numbers of autism cases (Fig. 4F). According to the literature, PSD-95, eIF4G1, G3BP2, NTRK2, Rap1GAP, and CPEB4, may potentially be degraded by autophagy, because they can be ubiquitinated [88–93] and autophagy degrades ubiquitinated proteins [94]. Thus, dysregulated PSD-95 and eIF4G1 may play strong roles in inducing autistic symptoms and behavioral deficits.

To validate the proteomic changes of PSD-95 and eIF4G1 in neurons, we performed Western blot analysis in cultured hippocampal neurons (Supplemental Fig. 10A). Consistent with the proteomics data, PSD-95 and eIF4G1 protein levels are significantly increased in *Fmr1* KO *vs*. WT hippocampal neurons and decreased by Rilmenidine (Supplemental Fig. 10B). Autophagy degrades ubiquitinated proteins [94] and downregulated autophagy in mouse brain is associated with accumulated ubiquitinated proteins [49]. Indeed, both total (Input) and ubiquitinated (IP) PSD-95 and eIF4G1 are increased in cultured hippocampal neurons when autophagy is inhibited by chemical inhibitors (Fig. 4G–J and Supplemental Fig. 11), indicating that PSD-95 and eIF4G1 are direct protein targets of autophagic protein degradation. Neurons from *Fmr1* KO mice exhibited markedly elevated total and ubiquitinated PSD-95 and eIF4G1 (Fig. 4G–J), indicating that ubiquitinated PSD-95 and eIF4G1 are not degraded efficiently in *Fmr1* KO neurons. Rilmenidine treatment significantly accelerated the degradation of PSD-95 and eIF4G1, shown by both decreased total and ubiquitinated PSD-95 and eIF4G1 proteins in *Fmr1* KO neurons (Fig. 4G–J, and Supplemental Fig. 11). In *Fmr1* KO mice with neuron-specific autophagy inhibition (*Atg7* knockout), Rilmenidine failed to reduce the levels of PSD-95 and eIF4G1 proteins in the hippocampus (Supplemental Fig. 12), further indicating that the Rilmenidine-induced degradation of PSD-95 and eIF4G1 requires autophagy. mRNA levels of PSD-95 and eIF4G1 were not affected by Rilmenidine, excluding the possibility that the altered protein levels are caused by changes in mRNAs (Supplemental Fig. 13). Altogether, these results indicate that eIF4G1 and PSD-95 are downstream targets of autophagy and may be responsible for the regulation on synaptic morphology. PSD-95 is a synaptic scaffolding protein crucially contributing to the stabilization and organization of postsynaptic structure, as many neurotransmitter receptors and postsynaptic cytoskeleton molecules are anchored to it [23, 95, 96]. When PSD-95 is upregulated, it leads to an overabundance of immature spines in hippocampal neurons [23, 95]. We previously reported that decreasing the PSD-95 levels by genetic manipulation of mTORC1 is associated with reduced spine density and increased maturation in hippocampal neurons [23]. Results from the current study indicated that Rilmenidine significantly reduces the PSD-95 level in the spine area (Supplemental Fig. 14), demonstrating that degrading PSD-95 to affect postsynaptic stability is one pivotal mechanism through which autophagy rescues spine deficits in FXS.

## Activation of autophagy degrades eIF4G1 to regulate actin dynamics in spines

**Fig. 5:**
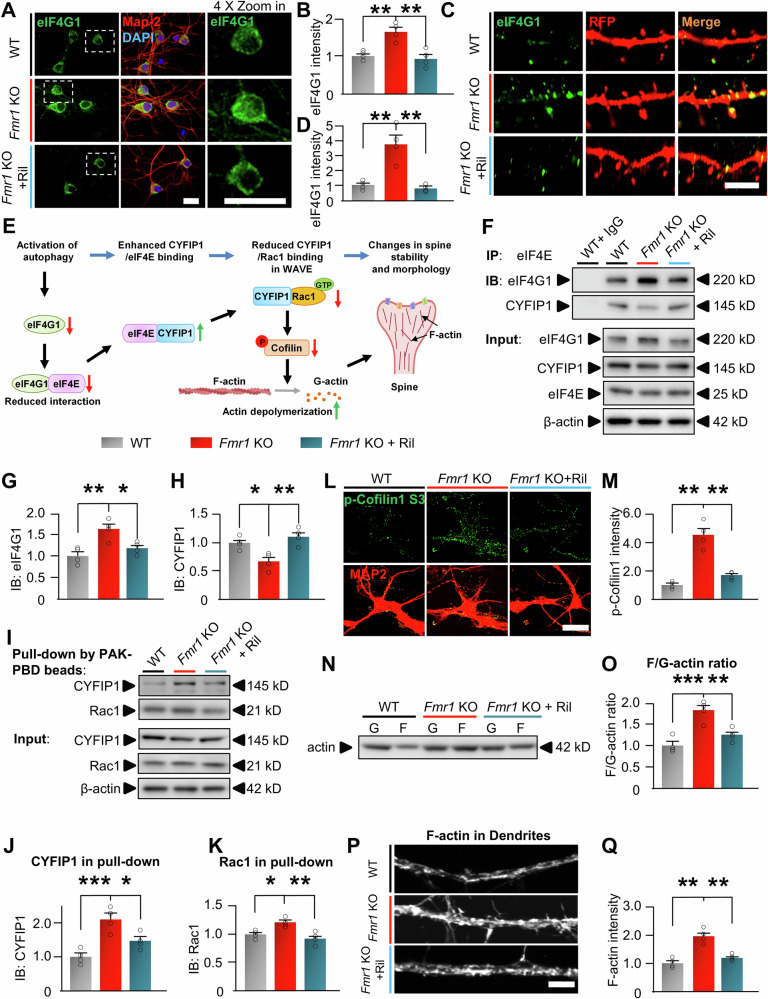
Autophagy regulates Cofilin1 activity and actin dynamics through eIF4G1. Primary neurons were cultured from hippocampus of WT and *Fmr1* KO mice and treated with Veh (DMSO) or Rilmenidine (10 µM) for 6 h. **A** Images show immunostaining of eIF4G1 together with MAP2 to mark neurons. eIF4G1 staining at higher magnification is shown in the right panels. Scale bar, 20 µm. **B** Summary bar graph shows fluorescent intensity of eIF4G1 puncta in A. **C** Cultured neurons were infected with a lentivirus expressing Syn-RFP to image dendrites. Images show immunolabeling of eIF4G1 in dendrites. Scale bar, 3 µm. **D** Summary bar graph shows fluorescent intensity of eIF4G1 puncta in C. **E** Schematic illustrating activation of autophagy degrades eIF4G1, reduces eIF4G1/eIF4E interaction, releases eIF4E to bind more CYFIP1, blocks the formation of WAVE complex, suppresses WAVE-induced Cofilin1 phosphorylation, and elevates F-actin depolymerization. **F** WT and *Fmr1* KO mice were injected with vehicle (as WT and *Fmr1* KO groups) or Rilmenidine (*Fmr1* KO + Ril). Protein lysates of hippocampal tissues were immunoprecipitated with an antibody to eIF4E. Lysates (Input) and immunoprecipitants (IP) were immunoblotted (IB) for eIF4E, eIF4G1, and CYFIP1. A naïve IgG antibody was used as negative control. **G, H** Bar graphs show summary data of F. **I** Protein lysates of hippocampus from treated mice were pulled down with PAK-PBD beads. Pulled-down proteins and lysates (input) were immunoblotted for CYFIP1 and Rac1. **J, K** Bar graphs show summary data of CYFIP1 and Rac1 in pull-down. **L** Primary hippocampal neurons from WT and *Fmr1* KO mice were treated with Veh (DMSO) or Rilmenidine (10 µM) for 6 h and then immunolabeled with p-Cofilin1(S3) and MAP2. Scale bar, 30 µm. **M** Summary bar graph shows fluorescent intensity of p-Cofilin1 puncta in L. **N** Synaptosome protein lysates from hippocampus of mice in labeled groups were assessed with F/G-actin ratio by Western blot. **O** Bar graph shows summarized ratio. **P** Primary hippocampal neurons from WT and *Fmr1* KO mice were treated with Veh or Rilmenidine (10 µM) for 6 h and imaged with F-actin. Scale bar, 3 µm. **Q** Summary bar graph shows fluorescent intensity of F-actin. Significance was calculated by ANOVA followed by a Tukey’s test. *p < 0.05, **p < 0.01. ***p < 0.001. β-actin was used as a loading control. Values were normalized to WT and reflect mean ± s.e.m. Each circle represents data from an individual mouse (5-week-old, n = 4 in each group) in G, H, J, K and O; or data from an independent culture (n = 4) in B, D, M, and Q.

We next explored the role of eIF4G1 in autophagic regulation of spine morphology. Our results indicate that the protein level of eIF4G1 in *Fmr1* KO neurons is increased in the soma (Fig. 5A, B), dendrites, and spines of neurons (Fig. 5C, D). Importantly, the increase in spine area is more significant than in soma (increased by 276% in spines *vs*. 43% in soma), suggesting that eIF4G1 may locally mediate autophagy’s regulation on spine morphology. The spine structure and morphology are majorly supported and determined by polymeric filamentous actin (F-actin), and Cofilin1 protein critically and directly catalyze depolymerization of F-actin to monomeric G-actin (actin dynamics) to destabilize spines and modify spine morphology [35, 97, 98]. Ras-related C3 botulinum toxin substrate 1 (Rac1) is an upstream regulator of Cofilin1 activity by inducing serine-3 phosphorylation of Cofilin1, primarily through p21-activated kinase (PAK)/LIM kinase, as well as through forming the Rac1-WAVE regulatory complex [30, 99]. eIF4G1 has been recently shown to crucially affect the assembly of Rac1-WAVE complex and downstream actin dynamics [31, 100]. The assembled Rac1-WAVE complex inactivates Cofilin1 by phosphorylating serine-3 residue and slows down the F-actin depolymerization [30, 99]. Thus, we hypothesized that activated autophagy degrades eIF4G1, reducing the interaction between eIF4G1 with eIF4E, to release eIF4E (Fig. 5E). The released eIF4E sequesters cytoplasmic FMRP-interacting protein 1 (CYFIP1), the essential component of Rac1–WAVE regulatory complex, and subsequently halt the assembly of Rac1-WAVE complex, which reduces Cofilin1 phosphorylation and enhances F-actin depolymerization (Fig. 5E) [31, 101]. Indeed, knockdown of eIF4G1 with shRNA in neurons significantly reduced the binding of eIF4G1 with eIF4E (Supplemental Fig. 15A–E), and enhanced the binding of eIF4E with CYFIP1(Supplemental Fig. 15A, F), indicating that manipulating eIF4G1 protein levels is able to affect the interaction of eIF4E/CYFIP1 to potentially regulate Rac1–WAVE regulatory complex. Thus, we next examined the binding of eIF4E with eIF4G1 or CYFIP1 through co-immunoprecipitation in hippocampal tissues of WT and *Fmr1* KO mice with/without Rilmenidine treatment (Fig. 5F). The input protein level of eIF4G1 in vehicle-treated *Fmr1* KO mice (*Fmr1* KO) is significantly higher than vehicle-treated WT mice (WT) (Supplemental Fig. 16A). Consistently, there is more eIF4G1 immunoprecipitated with eIF4E in *Fmr1* KO *vs*. WT mice, indicating an enhanced binding of eIF4E with eIF4G1 (Fig. 5G). Rilmenidine reduced the level of eIF4G1 in both input (Supplemental Fig. 16A) and immunoprecipitants with eIF4E (Fig. 5G), indicating that, by degrading eIF4G1, Rilmenidine treatment releases eIF4E from its binding with eIF4G1 in *Fmr1* KO mice. Subsequently, although there is a marginally lower level of total CYFIP1 in *Fmr1* KO mice (Supplemental Fig. 16B), more CYFIP1 proteins are co-immunoprecipitated with eIF4E in Rilmenidine treated *vs*. vehicle treated *Fmr1* KO mice (Fig. 5H), indicating that the released eIF4E from eIF4G1 binds and sequesters CYFIP1. We then used PAK-PBD beads to pull down Rac1-associated components of the Rac1-WAVE complex and examined whether sequestering CYFIP1 to eIF4E blocks the binding of CYFIP1 with Rac1 (Fig. 5I). The results show that more CYFIP1 was pulled down together with Rac1 in *Fmr1* KO *vs*. WT mice, and Rilmenidine significantly reduced the level of CYFIP1 interacting with Rac1 (Fig. 5J, K), indicating that there is less CYFIP1 forming the Rac1–WAVE regulatory complex in Rilmenidine treated *Fmr1* KO mice. As the assembly of Rac1–WAVE complex was suppressed, Rilmenidine treatment subsequently reduced Cofilin1 S-3 phosphorylation in *Fmr1* KO mice (Fig. 5L, M by immunostaining and Supplemental Fig. 17 by Western blot), indicating increased Cofilin1 activity. The F-actin/G-actin ratio in the hippocampal synaptic fraction is increased in *Fmr1* KO mice *vs*. WT (Fig. 5N, O). As a result of the increased Cofilin1 activity, Rilmenidine significantly reduced the F-actin/G-actin ratio, indicating that Rilmenidine accelerates F-actin depolymerization to affect spine morphology. The direct imaging of F-actin also confirmed that Rilmenidine reduced the F-actin levels in the dendritic area of cultured hippocampal *Fmr1* KO neurons (Fig. 5P, Q). In summary, our findings indicated that autophagic degradation of eIF4G1 elevates Cofilin1 activity and F-actin depolymerization to mediate the rescuing effects on spine density/morphology in FXS.

## Brain Rilmenidine infusion activates hippocampal autophagy and rescues deficits in FXS mice

Because Rilmenidine passes the blood-brain barrier freely as indicated by us and others [76], systemic *i.p*. injection of Rilmenidine activates autophagy in both peripheral tissues and the brain. To estimate the contribution of autophagy in the brain, especially in the hippocampus, to the rescue effects, we directly delivered Rilmenidine daily for 7 days to the lateral ventricles (close to the hippocampus) through cannulation (Supplemental Fig. 18A, B) [54]. Rilmenidine significantly reduced p62 accumulation, indicating activated autophagy in the hippocampus of *Fmr1* KO mice *vs. Fmr1* KO mice infused with vehicle (Supplemental Fig. 18C, D). Rilmenidine infusion also significantly reduced the protein levels of PSD-95 and eIF4G1 in the hippocampus of *Fmr1* KO mice (Supplemental Fig. 18C, E, F). Since the hippocampus is the primary brain region for cognition, we next examined whether central delivery of Rilmenidine rescues the impaired cognition of *Fmr1* KO mice. Consistent with the results from the systemic injection, Rilmenidine infusion to the lateral ventricles significantly increased the freezing reaction time of *Fmr1* KO mice, indicating improved cognition (Supplemental Fig. 18G). The infusion of Rilmenidine also improved the visual memory of *Fmr1* KO mice, demonstrated by increased interaction time with the novel object (Supplemental Fig. 18H, I). Mechanistic analysis indicated that Rilmenidine infusion corrected the increased F-actin/G-actin ratio in the hippocampal synaptic fraction of *Fmr1* KO mice, which implies that Rilmenidine infusion targets actin assembly for the rescue effect (Supplemental Fig. 18J, K). In general, our results demonstrate that central delivery of Rilmenidine activates autophagy and regulates actin assembly in the hippocampus of *Fmr1* KO mice, contributing to the rescued cognition.

## Effects of activation of autophagy on human FXS neurons

**Fig. 6:**
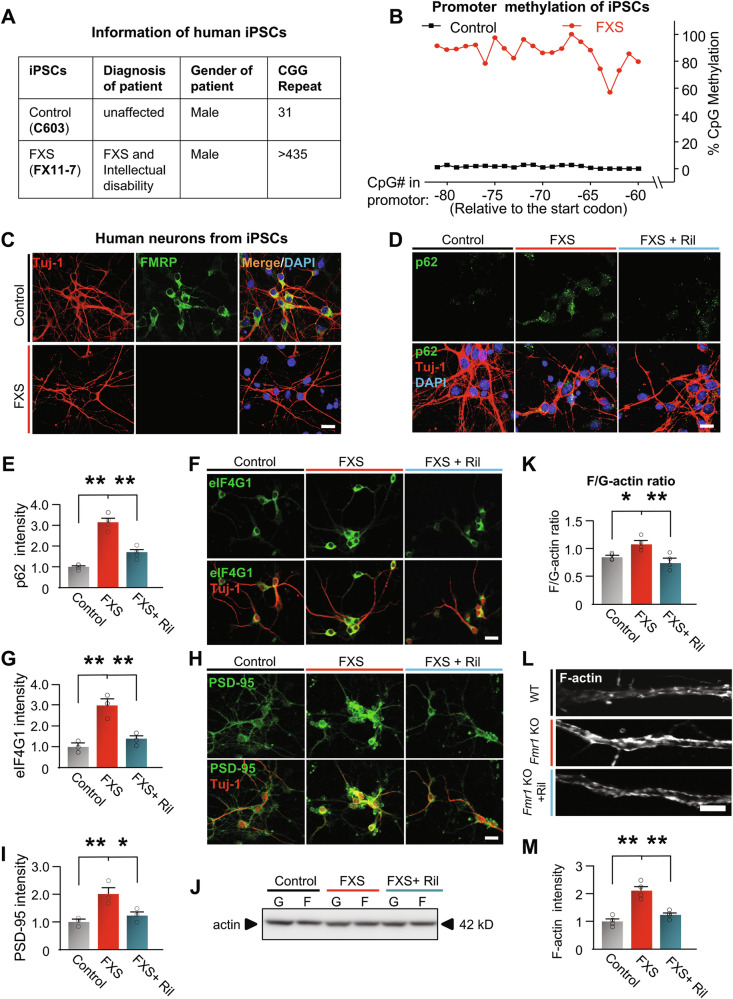
Activation of autophagy corrected the aberrant actin assembly in human FXS neurons. **A** Information of human iPSCs. **B** Average percentage of methylation on CpGs in the indicated promotor region of *Fmr1* gene. n = 3 samples in each group. **C** Neurons were differentiated from unaffected (control) and FXS iPSCs. FMRP expression was examined by immunostaining of FMRP and neuronal marker Tuj-1. Scale bar, 15 µm. **D** iPSCs-derived neurons were treated with DMSO as vehicle or Rilmenidine (Ril) (10 µM for 6 hr), and immunostained with p62 together with Tuj-1. Scale bar, 15 µm. **E** Summary bar graph shows fluorescent intensity of p62 puncta in D (normalized to the value of control). **F, H** iPSCs-derived neurons were treated with DMSO as vehicle or Rilmenidine (Ril) (10 µM for 6 hr), and immunostained with eIF4G1/PSD-95 together with Tuj-1. Scale bar, 20 µm. **G,** **I** Summary bar graphs show fluorescent intensities of eIF4G1 and PSD-95 puncta. **J** F/G-actin ratio was assessed by Western blot with lysates from iPSCs derived neurons. **K** Bar graph shows summary ratio of F/G (normalized to the value of control, n = 4 experiments). **L** iPSCs derived neurons were treated with Veh or Rilmenidine (10 µM) for 6 h and imaged with F-actin. Scale bar, 3 µm. **M** Summary bar graph shows fluorescent intensity of F-actin. Significance was calculated by ANOVA followed by a Tukey’s test. *p < 0.05, **p < 0.01. Values reflect mean ± s.e.m. Each circle in E, G, I, K and M represents data from an individual experiment.

Currently, there are still no clinical trials that can unambiguously show efficacy on FXS, mostly because of the gap between animal models and humans [3, 102]. Thus, we next assessed whether autophagy is downregulated in neurons derived from human FXS induced pluripotent stem cells (iPSCs), and neurons from an unaffected male individual were used as control (Fig. 6A) [55, 103]. The human FXS iPSCs were created from fibroblasts isolated from a male patient diagnosed with FXS (full mutation) and intellectual disability [55] (Fig. 6A). As epigenetic silencing of the *Fmr1* gene in FXS is caused by hypermethylation in its promoter region, we examined methylation on CpG islands in the *Fmr1* promoter of the iPSCs. The results show that FXS iPSCs have highly methylated CpG islands in the promotor, while the control iPSCs show nearly zero (Fig. 6B, Dataset S9). Assessment of *Fmr1* gene expression with immunostaining of FMRP indicated that there is no FMRP expression in neurons derived from FXS iPSCs, while the neurons from control iPSCs show strong FMRP expression (Fig. 6C). Consistent with *Fmr1* KO mice, neurons derived from FXS iPSCs show increased p62 accumulation *vs*. control, indicating downregulated autophagy (Fig. 6D, E). Rilmenidine treatment significantly reduced the p62 accumulation, indicating activated autophagy. Protein levels of PSD-95 and eIF4G1 are also increased in neurons derived from FXS iPSCs *vs*. control human neurons (Fig. 6F–I). Consistent with the mouse data, PSD-95 and eIF4G1 protein levels are significantly decreased by Rilmenidine (Fig. 6F–I). Further, neurons derived from FXS iPSCs show upregulated phosphorylation of Cofilin1 at S3 and increased F-actin/G-actin ratio *vs*. control, indicating dysregulated actin assembly (Supplemental Fig. 19, and Fig. 6J, K). When activating autophagy *via* Rilmenidine, both Cofilin1 phosphorylation and F-actin/G-actin ratio decreased to similar levels as control. F-actin imaging confirmed that Rilmenidine reduced the F-actin levels in the dendritic area of neurons derived from FXS iPSCs (Fig. 6L, M). We further validated our major findings with one more iPSC line (FX08-23) derived from a patient with FXS and diagnosis with intellectual disability (Supplemental Fig. 20A, B). [55, 103] The results indicated that Rilmenidine treatment significantly reduced p62 accumulation in neurons derived from FX08-23 iPSCs (Supplemental Fig. 20C, D). Protein levels of PSD-95 and eIF4G1 are increased in neurons derived from FX08-23 FXS iPSCs *vs*. control, which are significantly decreased by Rilmenidine (Supplemental Fig. 20E–H). F-actin imaging indicated that Rilmenidine significantly reduced the F-actin levels in dendritic area of neurons derived from FX08-23 iPSCs (Supplemental Fig. 20I–J). Thus, our results indicated that human FXS neurons show downregulated autophagy and dysregulated actin assembly, and activating autophagy corrected these defects.

## Discussion

Autophagy plays crucial roles in regulating synaptic structure, development, and plasticity and dysregulated autophagy is involved in many neurological disorders such as autism, stroke, and neurodegenerative diseases [23, 38, 39, 104–106]. Autophagy critically affects the stability and morphology of postsynaptic structures [23, 49, 50], yet the mechanism remains unclear. Activating autophagy with Rapamycin has been shown to activate the synaptic pruning and ameliorate the social deficits in *Tsc2* + /− ASD mice [49]. In this study, our findings revealed that activation of autophagy in the hippocampus of *Fmr1* KO mice rescued the aberrant spine morphology and improved cognition by affecting postsynaptic organization and actin dynamics. Currently, there is still no effective treatment for Fragile X in humans [102] and nearly all targeted treatments failed in clinical trials [3, 102]. One explanation is that, because FMRP influences hundreds of proteins and signal pathways, single targeted treatments are insufficient to rescue the complex dysregulated pathways and symptoms in FXS [102]. Thus, it is believed that treatments targeting multiple proteins and pathways are more likely to effectively reverse the multitude of changes in FXS brain [102]. Our findings revealed that, in hippocampus of a FXS mouse model, autophagy degrades multiple protein targets to affect synapse structures and functions on different levels. Among the 42 protein targets, PSD-95 is a scaffolding protein regulating postsynaptic organization and stability [95], and eIF4G1 regulates assembly of actin filaments, the major cytoskeletal elements of postsynaptic terminals [28, 101]. It has been reported that PSD-95 is ubiquitinated by the E3 ligase Mdm2 and degraded by proteasome, when dysregulated, causing increased spine density [95]. Our results show that ubiquitinated PSD-95 is also degraded by autophagy to affect spine stability. In the brains of an autistic mouse model caused by *Cullin3* gene deficiency, elevated eIF4G1 protein levels lead to increased spine density and impaired social behaviors [67]. In addition to PSD-95 and eIF4G1, it is possible that others of these 42 proteins, such as NTRK2 and CPEB4, are also targets of autophagic degradation and regulate synaptic functions.

Altered dendritic spine density and morphology are associated with many brain disorders, including neuropsychiatric diseases, autism, and neurodegenerative diseases [107]. However, the therapeutic strategy to correct spines in these diseases is still lacking. Dysregulation of autophagy has been extensively reported in neurodevelopmental and neurodegenerative disorders [23, 49, 104, 108–110]. It has also been well established that the cytoskeleton system plays critical roles in regulating autophagy through affecting autophagosome biogenesis, trafficking of autophagic components, and other processes [111]. In this study, we revealed a previously unappreciated pathway in which organization of actin cytoskeleton is regulated by autophagy through the degradation of eIF4G1 to affect the WAVE complex, which subsequently affects the stability and morphology of synapses. Several studies have recently reported that inhibition of eIF4G1 affects actin assembly by regulating the competition between eIF4E and Rac1 to bind CYFIP1 [28, 31]. The fact that *Cyfip1* heterozygote mice mimic key aspects of the Fragile X phenotype, such as overabundance of filopodial spines and exaggerated mGluR-LTD further indicates that eIF4G1-CYFIP1-Rac1-Cofilin1 pathway is critical for the regulation of spine morphology and functions [101]. Besides their role in regulating actin assembly, eIF4G1 and eIF4E are also critical for the initiation of translation [100]. Thus, reduced eIF4E/eIF4G1 binding by Rilmenidine may also affect spine morphology by interfering with protein translation. Pharmacologically inhibiting the interaction between eIF4G1/eIF4E suppressed translation and has been used to affect spine morphology in autism mouse models [67, 112]. Our results show that activation of autophagy by Rilmenidine reduced protein synthesis by ~20% in primary hippocampal *Fmr1* KO neurons (Supplemental Fig. 21). Thus, autophagy may affect spine morphology by regulating both actin assembly and protein translation. Rilmenidine is a classical imidazoline type 1 receptor (I1R/IRAS/Nischarin) agonist in mammals [113]. Nischarin can bind activated PAK1, the Rac1 effector, to inhibit Rac1/PAK1 activation [114]. Since activated Rac1/PAK1 phosphorylates and activates LIM kinase, which directly phosphorylates and inactivates Cofilin1, Rilmenidine may also modulate Cofilin1 and F-actin assembly through Rac1/PAK1 signaling. In addition, Rilmenidine has been reported to stimulate the proapoptotic protein Bax and induce the perturbation of the mitochondrial pathway [115]. Mitochondrial ATP synthase leak in synapses is causally related to the aberrant Fragile X associated spine morphology and behaviors [22]. Thus, potential effects of Rilmenidine on synaptic mitochondrial function should also be considered.

Our results demonstrated that activating autophagy through Rilmenidine treatment largely rescued the cognitive deficits in *Fmr1* KO FXS mouse model. However, loss of FMRP in FXS impacts ~1000 neuronal mRNAs and complex signal pathways which are critical to neural development, synaptic plasticity, and dendritic spine architecture [1, 8–12]. Many of these pathways independently and synergistically contribute to the diverse phenotypes and deficits observed in FXS. Thus, it is important to emphasize that dysfunctional autophagy in hippocampus is unlikely to be solely responsible for all deficits in FXS. Indeed, we observed that Rilmenidine treatment failed to rescue the deficits of nest building and open field tests associated with *Fmr1* KO mice. This suggests that effects of Rilmenidine treatment and activated autophagy in other brain regions and related behavioral deficits need to be further examined in future studies. Systemic administration of Rilmenidine may affect other tissues, such as heart and kidney, in addition to brain. Clinical trials show long-term administration of Rilmenidine is effective in both reducing left ventricular mass and decreasing blood pressure by decreasing vascular resistance [116, 117]. In the kidney, clinical investigation reveals Rilmenidine reduces microalbuminuria in hypertensive type-2 diabetic patients, as well as preserve renal function during stress-induced high blood pressure [118, 119]. Although our results indicated that there was no significant effect of Rilmenidine on body weight and growth of mice (Supplemental Fig. 4A, B), caution still needs to be exercised when considering Rilmenidine as a possible treatment option. To further address translational relevance, we verified dysfunctional autophagy and downstream pathways with human neurons derived from iPSCs generated from two individuals with FXS. While this provides initial validation in a human system, the use of two iPSC lines remains a limitation, as it does not fully capture the variability across individuals with FXS.

In summary, our study identified a new role of autophagy in actin assembly, spine morphology, and cognitive deficits in *Fmr1* KO mice. These findings identify autophagy as a therapeutic target for Fragile X syndrome. Dysregulated autophagy and its upstream regulator, mTORC1 signaling are implicated not only in FXS, but also in mouse models of other autism spectrum disorders, including Rett syndrome, *TSC, PTEN*, and 16p11.2 deletion [49, 120–122]. Sulzer and colleagues show that overactivated mTOR suppresses autophagy in the brain of *Tsc1*^*+/−*^ and *Tsc2*^*+/−*^ mice, and that reduced autophagy impaired spine pruning of spines of cortical layer V pyramidal neurons and induced autistic behaviors [49]. Thus, findings in the present study suggest components of the autophagy pathway may represent promising therapeutic targets, not only for Fragile X syndrome, but also other ASDs.

## Supplementary information

Supplemental Materials

## Data Availability

Source Data for all figures are provided with the paper, and reagents and all other data are available from the corresponding author upon reasonable request. The datasets have been stored in Open Science Framework (OSF) (https://osf.io), associated with project “Autophagy Fragile X syndrome. MP DATA”.

## References

[1] Bhakar AL, Dolen G, Bear MF. The pathophysiology of fragile X (and what it teaches us about synapses). Annu Rev Neurosci. 2012;35:417–43. 22483044 10.1146/annurev-neuro-060909-153138 PMC4327822

[2] Bagni C, Tassone F, Neri G, Hagerman R. Fragile X syndrome: causes, diagnosis, mechanisms, and therapeutics. J Clin Invest. 2012;122:4314–22. 23202739 10.1172/JCI63141 PMC3533539

[3] Berry-Kravis EM, Lindemann L, Jonch AE, Apostol G, Bear MF, Carpenter RL, et al. Drug development for neurodevelopmental disorders: lessons learned from fragile X syndrome. Nat Rev Drug Discov. 2018;17:280–99. 29217836 10.1038/nrd.2017.221 PMC6904225

[4] Penagarikano O, Mulle JG, Warren ST. The pathophysiology of fragile x syndrome. Annu Rev Genomics Hum Genet. 2007;8:109–29. 17477822 10.1146/annurev.genom.8.080706.092249

[5] Richter JD, Bassell GJ, Klann E. Dysregulation and restoration of translational homeostasis in fragile X syndrome. Nat Rev Neurosci. 2015;16:595–605. 26350240 10.1038/nrn4001 PMC4688896

[6] Rodriguez CM, Wright SE, Kearse MG, Haenfler JM, Flores BN, Liu Y, et al. A native function for RAN translation and CGG repeats in regulating fragile X protein synthesis. Nat Neurosci. 2020;23:386–97. 32066985 10.1038/s41593-020-0590-1 PMC7668390

[7] Zhan X, Asmara H, Cheng N, Sahu G, Sanchez E, Zhang FX, et al. FMRP(1-297)-tat restores ion channel and synaptic function in a model of Fragile X syndrome. Nat Commun. 2020;11:2755. 32488011 10.1038/s41467-020-16250-4 PMC7265297

[8] Kelleher RJ 3rd, Bear MF. The autistic neuron: troubled translation? Cell. 2008;135:401–6. 18984149 10.1016/j.cell.2008.10.017

[9] Darnell JC, Klann E. The translation of translational control by FMRP: therapeutic targets for FXS. Nat Neurosci. 2013;16:1530–6. 23584741 10.1038/nn.3379 PMC3999698

[10] Gross C, Chang CW, Kelly SM, Bhattacharya A, McBride SM, Danielson SW, et al. Increased expression of the PI3K enhancer PIKE mediates deficits in synaptic plasticity and behavior in fragile X syndrome. Cell Rep. 2015;11:727–36. 25921541 10.1016/j.celrep.2015.03.060 PMC4418204

[11] Vita DJ, Meier CJ, Broadie K. Neuronal fragile X mental retardation protein activates glial insulin receptor mediated PDF-Tri neuron developmental clearance. Nat Commun. 2021;12:1160. 33608547 10.1038/s41467-021-21429-4 PMC7896095

[12] Monday HR, Kharod SC, Yoon YJ, Singer RH, Castillo PE. Presynaptic FMRP and local protein synthesis support structural and functional plasticity of glutamatergic axon terminals. Neuron. 2022;110:2588–2606.e2586. 35728596 10.1016/j.neuron.2022.05.024 PMC9391299

[13] He Q, Arroyo ED, Smukowski SN, Xu J, Piochon C, Savas JN, et al. Critical period inhibition of NKCC1 rectifies synapse plasticity in the somatosensory cortex and restores adult tactile response maps in fragile X mice. Mol Psychiatry. 2019;24:1732–47. 29703945 10.1038/s41380-018-0048-y PMC6204122

[14] Goel A, Cantu DA, Guilfoyle J, Chaudhari GR, Newadkar A, Todisco B, et al. Impaired perceptual learning in a mouse model of Fragile X syndrome is mediated by parvalbumin neuron dysfunction and is reversible. Nat Neurosci. 2018;21:1404–11. 30250263 10.1038/s41593-018-0231-0 PMC6161491

[15] Seo SS, Louros SR, Anstey N, Gonzalez-Lozano MA, Harper CB, Verity NC, et al. Excess ribosomal protein production unbalances translation in a model of Fragile X syndrome. Nat Commun. 2022;13:3236. 35688821 10.1038/s41467-022-30979-0 PMC9187743

[16] Bhaskaran AA, Gauvrit T, Vyas Y, Bony G, Ginger M, Frick A. Endogenous noise of neocortical neurons correlates with atypical sensory response variability in the Fmr1(-/y) mouse model of autism. Nat Commun. 2023;14:7905. 38036566 10.1038/s41467-023-43777-z PMC10689491

[17] Brown MR, Kronengold J, Gazula VR, Chen Y, Strumbos JG, Sigworth FJ, et al. Fragile X mental retardation protein controls gating of the sodium-activated potassium channel Slack. Nat Neurosci. 2010;13:819–21. 20512134 10.1038/nn.2563 PMC2893252

[18] Berry KP, Nedivi E. Spine dynamics: are they all the same? Neuron. 2017;96:43–55. 28957675 10.1016/j.neuron.2017.08.008 PMC5661952

[19] Irwin SA, Patel B, Idupulapati M, Harris JB, Crisostomo RA, Larsen BP, et al. Abnormal dendritic spine characteristics in the temporal and visual cortices of patients with fragile-X syndrome: a quantitative examination. Am J Med Genet. 2001;98:161–7. 11223852 10.1002/1096-8628(20010115)98:2<161::aid-ajmg1025>3.0.co;2-b

[20] Nimchinsky EA, Oberlander AM, Svoboda K. Abnormal development of dendritic spines in FMR1 knock-out mice. J Neurosci. 2001;21:5139–46. 11438589 10.1523/JNEUROSCI.21-14-05139.2001 PMC6762842

[21] Booker SA, Domanski APF, Dando OR, Jackson AD, Isaac JTR, Hardingham GE, et al. Altered dendritic spine function and integration in a mouse model of fragile X syndrome. Nat Commun. 2019;10:4813. 31645626 10.1038/s41467-019-11891-6 PMC6811549

[22] Licznerski P, Park HA, Rolyan H, Chen R, Mnatsakanyan N, Miranda P, et al. ATP synthase c-Subunit leak causes aberrant cellular metabolism in Fragile X syndrome. Cell. 2020;182:1170–1185.e1179. 32795412 10.1016/j.cell.2020.07.008 PMC7484101

[23] Yan J, Porch MW, Court-Vazquez B, Bennett MVL, Zukin RS. Activation of autophagy rescues synaptic and cognitive deficits in fragile X mice. Proc Natl Acad Sci USA. 2018;115:E9707–E9716. 30242133 10.1073/pnas.1808247115 PMC6187122

[24] Dolan BM, Duron SG, Campbell DA, Vollrath B, Shankaranarayana Rao BS, Ko HY, et al. Rescue of fragile X syndrome phenotypes in Fmr1 KO mice by the small-molecule PAK inhibitor FRAX486. Proc Natl Acad Sci USA. 2013;110:5671–6. 23509247 10.1073/pnas.1219383110 PMC3619302

[25] Udagawa T, Farny NG, Jakovcevski M, Kaphzan H, Alarcon JM, Anilkumar S, et al. Genetic and acute CPEB1 depletion ameliorate fragile X pathophysiology. Nat Med. 2013;19:1473–7. 24141422 10.1038/nm.3353 PMC3823751

[26] Gantois I, Khoutorsky A, Popic J, Aguilar-Valles A, Freemantle E, Cao R, et al. Metformin ameliorates core deficits in a mouse model of fragile X syndrome. Nat Med. 2017;23:674–7. 28504725 10.1038/nm.4335

[27] Busquets-Garcia A, Gomis-Gonzalez M, Guegan T, Agustin-Pavon C, Pastor A, Mato S, et al. Targeting the endocannabinoid system in the treatment of fragile X syndrome. Nat Med. 2013;19:603–7. 23542787 10.1038/nm.3127

[28] Mercaldo V, Vidimova B, Gastaldo D, Fernandez E, Lo AC, Cencelli G, et al. Altered striatal actin dynamics drives behavioral inflexibility in a mouse model of fragile X syndrome. Neuron. 2023;111:1760–1775.e1768. 36996810 10.1016/j.neuron.2023.03.008

[29] Shen M, Wang F, Li M, Sah N, Stockton ME, Tidei JJ, et al. Reduced mitochondrial fusion and Huntingtin levels contribute to impaired dendritic maturation and behavioral deficits in Fmr1-mutant mice. Nat Neurosci. 2019;22:386–400. 30742117 10.1038/s41593-019-0338-y PMC6556892

[30] Pyronneau A, He Q, Hwang JY, Porch M, Contractor A, Zukin RS. Aberrant Rac1-cofilin signaling mediates defects in dendritic spines, synaptic function, and sensory perception in fragile X syndrome. Sci Signal. 2017;10:eaan0852. 29114038 10.1126/scisignal.aan0852 PMC5988355

[31] Santini E, Huynh TN, Longo F, Koo SY, Mojica E, D’Andrea L, et al. Reducing eIF4E-eIF4G interactions restores the balance between protein synthesis and actin dynamics in fragile X syndrome model mice. Sci Signal. 2017;10:eaan0665. 29114037 10.1126/scisignal.aan0665 PMC5858943

[32] Cingolani LA, Goda Y. Actin in action: the interplay between the actin cytoskeleton and synaptic efficacy. Nat Rev Neurosci. 2008;9:344–56. 18425089 10.1038/nrn2373

[33] Hotulainen P, Hoogenraad CC. Actin in dendritic spines: connecting dynamics to function. J Cell Biol. 2010;189:619–29. 20457765 10.1083/jcb.201003008 PMC2872912

[34] Forrest MP, Parnell E, Penzes P. Dendritic structural plasticity and neuropsychiatric disease. Nat Rev Neurosci. 2018;19:215–34. 29545546 10.1038/nrn.2018.16 PMC6442683

[35] Pontrello CG, Sun MY, Lin A, Fiacco TA, DeFea KA, Ethell IM. Cofilin under control of beta-arrestin-2 in NMDA-dependent dendritic spine plasticity, long-term depression (LTD), and learning. Proc Natl Acad Sci USA. 2012;109:E442–451. 22308427 10.1073/pnas.1118803109 PMC3289389

[36] Harris H, Rubinsztein DC. Control of autophagy as a therapy for neurodegenerative disease. Nat Rev Neurol. 2011;8:108–17. 22187000 10.1038/nrneurol.2011.200

[37] Nixon RA. The role of autophagy in neurodegenerative disease. Nat Med. 2013;19:983–97. 23921753 10.1038/nm.3232

[38] Mizushima N, Levine B, Cuervo AM, Klionsky DJ. Autophagy fights disease through cellular self-digestion. Nature. 2008;451:1069–75. 18305538 10.1038/nature06639 PMC2670399

[39] Shehata M, Matsumura H, Okubo-Suzuki R, Ohkawa N, Inokuchi K. Neuronal stimulation induces autophagy in hippocampal neurons that is involved in AMPA receptor degradation after chemical long-term depression. J Neurosci. 2012;32:10413–22. 22836274 10.1523/JNEUROSCI.4533-11.2012 PMC6703735

[40] Linda K, Lewerissa EI, Verboven AHA, Gabriele M, Frega M, Klein Gunnewiek TM, et al. Imbalanced autophagy causes synaptic deficits in a human model for neurodevelopmental disorders. Autophagy. 2022;18:423–42. 34286667 10.1080/15548627.2021.1936777 PMC8942553

[41] Compans B, Camus C, Kallergi E, Sposini S, Martineau M, Butler C, et al. NMDAR-dependent long-term depression is associated with increased short term plasticity through autophagy mediated loss of PSD-95. Nat Commun. 2021;12:2849. 33990590 10.1038/s41467-021-23133-9 PMC8121912

[42] Kallergi E, Daskalaki AD, Kolaxi A, Camus C, Ioannou E, Mercaldo V, et al. Dendritic autophagy degrades postsynaptic proteins and is required for long-term synaptic depression in mice. Nat Commun. 2022;13:680. 35115539 10.1038/s41467-022-28301-z PMC8814153

[43] Shen H, Zhu H, Panja D, Gu Q, Li Z. Autophagy controls the induction and developmental decline of NMDAR-LTD through endocytic recycling. Nat Commun. 2020;11:2979. 32532981 10.1038/s41467-020-16794-5 PMC7293213

[44] Nikoletopoulou V, Tavernarakis N. Regulation and roles of autophagy at synapses. Trends Cell Biol. 2018;28:646–61. 29731196 10.1016/j.tcb.2018.03.006

[45] Negrete-Hurtado A, Overhoff M, Bera S, De Bruyckere E, Schätzmüller K, Kye MJ, et al. Autophagy lipidation machinery regulates axonal microtubule dynamics but is dispensable for survival of mammalian neurons. Nat Commun. 2020;11:1535. 32210230 10.1038/s41467-020-15287-9 PMC7093409

[46] Crawley O, Opperman KJ, Desbois M, Adrados I, Borgen MA, Giles AC, et al. Autophagy is inhibited by ubiquitin ligase activity in the nervous system. Nat Commun. 2019;10:5017. 31676756 10.1038/s41467-019-12804-3 PMC6825199

[47] Kiral FR, Linneweber GA, Mathejczyk T, Georgiev SV, Wernet MF, Hassan BA, et al. Autophagy-dependent filopodial kinetics restrict synaptic partner choice during Drosophila brain wiring. Nat Commun. 2020;11:1325. 32165611 10.1038/s41467-020-14781-4 PMC7067798

[48] Bagni C, Zukin RS. A synaptic perspective of Fragile X syndrome and autism spectrum disorders. Neuron. 2019;101:1070–88. 30897358 10.1016/j.neuron.2019.02.041 PMC9628679

[49] Tang G, Gudsnuk K, Kuo SH, Cotrina ML, Rosoklija G, Sosunov A, et al. Loss of mTOR-dependent macroautophagy causes autistic-like synaptic pruning deficits. Neuron. 2014;83:1131–43. 25155956 10.1016/j.neuron.2014.07.040 PMC4159743

[50] Nikoletopoulou V, Sidiropoulou K, Kallergi E, Dalezios Y, Tavernarakis N. Modulation of autophagy by BDNF underlies synaptic plasticity. Cell Metab. 2017;26:230–242.e235. 28683289 10.1016/j.cmet.2017.06.005

[51] Guo Y, Shen M, Dong Q, Mendez-Albelo NM, Huang SX, Sirois CL, et al. Elevated levels of FMRP-target MAP1B impair human and mouse neuronal development and mouse social behaviors via autophagy pathway. Nat Commun. 2023;14:3801. 37365192 10.1038/s41467-023-39337-0 PMC10293283

[52] Spencer CM, Serysheva E, Yuva-Paylor LA, Oostra BA, Nelson DL, Paylor R. Exaggerated behavioral phenotypes in Fmr1/Fxr2 double knockout mice reveal a functional genetic interaction between Fragile X-related proteins. Hum Mol Genet. 2006;15:1984–94. 16675531 10.1093/hmg/ddl121

[53] Miyawaki T, Ofengeim D, Noh KM, Latuszek-Barrantes A, Hemmings BA, Follenzi A, et al. The endogenous inhibitor of Akt, CTMP, is critical to ischemia-induced neuronal death. Nat Neurosci. 2009;12:618–26. 19349976 10.1038/nn.2299 PMC2724841

[54] Yan J, Zhang H, Yin Y, Li J, Tang Y, Purkayastha S, et al. Obesity- and aging-induced excess of central transforming growth factor-β potentiates diabetic development via an RNA stress response. Nat Med. 2014;20:1001. 25086906 10.1038/nm.3616 PMC4167789

[55] Doers ME, Musser MT, Nichol R, Berndt ER, Baker M, Gomez TM, et al. iPSC-derived forebrain neurons from FXS individuals show defects in initial neurite outgrowth. Stem Cells Dev. 2014;23:1777–87. 24654675 10.1089/scd.2014.0030 PMC4103262

[56] Chambers SM, Fasano CA, Papapetrou EP, Tomishima M, Sadelain M, Studer L. Highly efficient neural conversion of human ES and iPS cells by dual inhibition of SMAD signaling. Nat Biotechnol. 2009;27:275–80. 19252484 10.1038/nbt.1529 PMC2756723

[57] Li M, Zhao H, Ananiev GE, Musser MT, Ness KH, Maglaque DL, et al. Establishment of reporter lines for detecting Fragile X mental retardation (FMR1) gene reactivation in human neural cells. Stem Cells. 2017;35:158–69. 27422057 10.1002/stem.2463 PMC5195860

[58] Autar K, Guo X, Rumsey JW, Long CJ, Akanda N, Jackson M, et al. A functional hiPSC-cortical neuron differentiation and maturation model and its application to neurological disorders. Stem Cell Reports. 2022;17:96–109. 34942087 10.1016/j.stemcr.2021.11.009 PMC8758945

[59] Tost J, Dunker J, Gut IG. Analysis and quantification of multiple methylation variable positions in CpG islands by pyrosequencing. Biotechniques. 2003;35:152–6. 12866415 10.2144/03351md02

[60] Bhattacharya A, Kaphzan H, Alvarez-Dieppa AC, Murphy JP, Pierre P, Klann E. Genetic removal of p70 S6 kinase 1 corrects molecular, synaptic, and behavioral phenotypes in fragile X syndrome mice. Neuron. 2012;76:325–37. 23083736 10.1016/j.neuron.2012.07.022 PMC3479445

[61] Li J, Tang Y, Cai D. IKKbeta/NF-kappaB disrupts adult hypothalamic neural stem cells to mediate a neurodegenerative mechanism of dietary obesity and pre-diabetes. Nat Cell Biol. 2012;14:999–1012. 22940906 10.1038/ncb2562 PMC3463771

[62] Dunkley PR, Jarvie PE, Robinson PJ. A rapid percoll gradient procedure for preparation of synaptosomes. Nat Protoc. 2008;3:1718–28. 18927557 10.1038/nprot.2008.171

[63] Zhang Y, Helm JS, Senatore A, Spafford JD, Kaczmarek LK, Jonas EA. PKC-induced intracellular trafficking of Ca(V)2 precedes its rapid recruitment to the plasma membrane. J Neurosci. 2008;28:2601–12. 18322103 10.1523/JNEUROSCI.4314-07 PMC2830008

[64] Rodenas-Ruano A, Chavez AE, Cossio MJ, Castillo PE, Zukin RS. REST-dependent epigenetic remodeling promotes the developmental switch in synaptic NMDA receptors. Nat Neurosci. 2012;15:1382–90. 22960932 10.1038/nn.3214 PMC3501125

[65] Huang W, Zhu PJ, Zhang S, Zhou H, Stoica L, Galiano M, et al. mTORC2 controls actin polymerization required for consolidation of long-term memory. Nat Neurosci. 2013;16:441–8. 23455608 10.1038/nn.3351 PMC3615448

[66] Schmidt EK, Clavarino G, Ceppi M, Pierre P. SUnSET, a nonradioactive method to monitor protein synthesis. Nat Methods. 2009;6:275–7. 19305406 10.1038/nmeth.1314

[67] Dong Z, Chen W, Chen C, Wang H, Cui W, Tan Z, et al. CUL3 deficiency causes social deficits and anxiety-like behaviors by impairing excitation-inhibition balance through the promotion of cap-dependent translation. Neuron. 2020;105:475–490.e476. 31780330 10.1016/j.neuron.2019.10.035 PMC7007399

[68] Stoppel LJ, Kazdoba TM, Schaffler MD, Preza AR, Heynen A, Crawley JN, et al. R-Baclofen reverses cognitive deficits and improves social interactions in two lines of 16p11.2 deletion mice. Neuropsychopharmacology. 2018;43:513–24. 28984295 10.1038/npp.2017.236 PMC5770771

[69] Deacon RM. Assessing nest building in mice. Nat Protoc. 2006;1:1117–9. 17406392 10.1038/nprot.2006.170

[70] Gao XH, Li L, Parisien M, Wu J, Bederman I, Gao Z, et al. Discovery of a redox thiol switch: implications for cellular energy metabolism. Mol Cell Proteomics. 2020;19:852–70. 32132231 10.1074/mcp.RA119.001910 PMC7196587

[71] Vodicka P, Lim J, Williams DT, Kegel KB, Chase K, Park H, et al. Assessment of chloroquine treatment for modulating autophagy flux in brain of WT and HD mice. J Huntingtons Dis. 2014;3:159–74. 25062859 10.3233/JHD-130081

[72] Roncaglia P, Martone ME, Hill DP, Berardini TZ, Foulger RE, Imam FT, et al. The Gene Ontology (GO) cellular component ontology: integration with SAO (Subcellular Anatomy Ontology) and other recent developments. J Biomed Semantics. 2013;4:20. 24093723 10.1186/2041-1480-4-20 PMC3852282

[73] Contractor A, Klyachko VA, Portera-Cailliau C. Altered neuronal and circuit excitability in Fragile X syndrome. Neuron. 2015;87:699–715. 26291156 10.1016/j.neuron.2015.06.017 PMC4545495

[74] Koopmans F, van Nierop P, Andres-Alonso M, Byrnes A, Cijsouw T, Coba MP, et al. SynGO: an evidence-based, expert-curated knowledge base for the synapse. Neuron. 2019;103:217–234.e214. 31171447 10.1016/j.neuron.2019.05.002 PMC6764089

[75] Rubinsztein DC, Codogno P, Levine B. Autophagy modulation as a potential therapeutic target for diverse diseases. Nat Rev Drug Discov. 2012;11:709–30. 22935804 10.1038/nrd3802 PMC3518431

[76] Underwood BR, Green-Thompson ZW, Pugh PJ, Lazic SE, Mason SL, Griffin J, et al. An open-label study to assess the feasibility and tolerability of rilmenidine for the treatment of Huntington’s disease. J Neurol. 2017;264:2457–63. 29075837 10.1007/s00415-017-8647-0 PMC5688221

[77] Bennett DF, Goyala A, Statzer C, Beckett CW, Tyshkovskiy A, Gladyshev VN, et al. Rilmenidine extends lifespan and healthspan in Caenorhabditis elegans via a nischarin I1-imidazoline receptor. Aging Cell. 2023;22:e13774. 36670049 10.1111/acel.13774 PMC9924948

[78] Choi DH, Kim DH, Park YG, Chun BG, Choi SH. Protective effects of rilmenidine and AGN 192403 on oxidative cytotoxicity and mitochondrial inhibitor-induced cytotoxicity in astrocytes. Free Radic Biol Med. 2002;33:1321–33. 12419464 10.1016/s0891-5849(02)01041-9

[79] Rose C, Menzies FM, Renna M, Acevedo-Arozena A, Corrochano S, Sadiq O, et al. Rilmenidine attenuates toxicity of polyglutamine expansions in a mouse model of Huntington’s disease. Hum Mol Genet. 2010;19:2144–53. 20190273 10.1093/hmg/ddq093 PMC2865373

[80] Perera ND, Sheean RK, Lau CL, Shin YS, Beart PM, Horne MK, et al. Rilmenidine promotes MTOR-independent autophagy in the mutant SOD1 mouse model of amyotrophic lateral sclerosis without slowing disease progression. Autophagy. 2018;14:534–51. 28980850 10.1080/15548627.2017.1385674 PMC5915012

[81] Klionsky DJ, Abdel-Aziz AK, Abdelfatah S, Abdellatif M, Abdoli A, Abel S, et al. Guidelines for the use and interpretation of assays for monitoring autophagy (4th edition)(1). Autophagy. 2021;17:1–382. 33634751 10.1080/15548627.2020.1797280 PMC7996087

[82] Palmer JE, Wilson N, Son SM, Obrocki P, Wrobel L, Rob M, et al. Autophagy, aging, and age-related neurodegeneration. Neuron. 2024. 10.1016/j.neuron.2024.09.015 39406236

[83] Fleming A, Bourdenx M, Fujimaki M, Karabiyik C, Krause GJ, Lopez A, et al. The different autophagy degradation pathways and neurodegeneration. Neuron. 2022;110:935–66. 35134347 10.1016/j.neuron.2022.01.017 PMC8930707

[84] McCary LM, Roberts JE. Early identification of autism in fragile X syndrome: a review. J Intellect Disabil Res. 2013;57:803–14. 22974167 10.1111/j.1365-2788.2012.01609.x PMC4023162

[85] Luscher C, Huber KM. Group 1 mGluR-dependent synaptic long-term depression: mechanisms and implications for circuitry and disease. Neuron. 2010;65:445–59. 20188650 10.1016/j.neuron.2010.01.016 PMC2841961

[86] Kazdoba TM, Leach PT, Silverman JL, Crawley JN. Modeling fragile X syndrome in the Fmr1 knockout mouse. Intractable Rare Dis Res. 2014;3:118–33. 25606362 10.5582/irdr.2014.01024 PMC4298642

[87] Levine B, Packer M, Codogno P. Development of autophagy inducers in clinical medicine. J Clin Invest. 2015;125:14–24. 25654546 10.1172/JCI73938 PMC4382267

[88] Wang N, Li T, Liu W, Lin J, Zhang K, Li Z, et al. USP7- and PRMT5-dependent G3BP2 stabilization drives de novo lipogenesis and tumorigenesis of HNSC. Cell Death Dis. 2023;14:182. 36878903 10.1038/s41419-023-05706-2 PMC9988876

[89] Alard A, Fabre B, Anesia R, Marboeuf C, Pierre P, Susini C, et al. NAD(P)H quinone-oxydoreductase 1 protects eukaryotic translation initiation factor 4GI from degradation by the proteasome. Mol Cell Biol. 2010;30:1097–105. 20028737 10.1128/MCB.00868-09 PMC2815573

[90] Colledge M, Snyder EM, Crozier RA, Soderling JA, Jin Y, Langeberg LK, et al. Ubiquitination regulates PSD-95 degradation and AMPA receptor surface expression. Neuron. 2003;40:595–607. 14642282 10.1016/s0896-6273(03)00687-1 PMC3963808

[91] Makkerh JP, Ceni C, Auld DS, Vaillancourt F, Dorval G, Barker PA. p75 neurotrophin receptor reduces ligand-induced Trk receptor ubiquitination and delays Trk receptor internalization and degradation. EMBO Rep. 2005;6:936–41. 16113645 10.1038/sj.embor.7400503 PMC1369184

[92] Wang Y, Xie Y, Sun B, Guo Y, Song L, Mohammednur DE, et al. The degradation of Rap1GAP via E6AP-mediated ubiquitin-proteasome pathway is associated with HPV16/18-infection in cervical cancer cells. Infect Agent Cancer. 2021;16:71. 34952616 10.1186/s13027-021-00409-9 PMC8710002

[93] Liu Z, Gu S, Wu K, Li L, Dong C, Wang W, et al. CircRNA-DOPEY2 enhances the chemosensitivity of esophageal cancer cells by inhibiting CPEB4-mediated Mcl-1 translation. J Exp Clin Cancer Res. 2021;40:361. 34781999 10.1186/s13046-021-02149-5 PMC8591801

[94] Zhao J, Zhai B, Gygi SP, Goldberg AL. mTOR inhibition activates overall protein degradation by the ubiquitin proteasome system as well as by autophagy. Proc Natl Acad Sci USA. 2015;112:15790–7. 26669439 10.1073/pnas.1521919112 PMC4703015

[95] Tsai NP, Wilkerson JR, Guo W, Maksimova MA, DeMartino GN, Cowan CW, et al. Multiple autism-linked genes mediate synapse elimination via proteasomal degradation of a synaptic scaffold PSD-95. Cell. 2012;151:1581–94. 23260144 10.1016/j.cell.2012.11.040 PMC3530171

[96] Matt L, Kim K, Hergarden AC, Patriarchi T, Malik ZA, Park DK, et al. alpha-Actinin Anchors PSD-95 at Postsynaptic Sites. Neuron. 2018;97:1094–1109.e1099. 29429936 10.1016/j.neuron.2018.01.036 PMC5963734

[97] Zhou Q, Homma KJ, Poo MM. Shrinkage of dendritic spines associated with long-term depression of hippocampal synapses. Neuron. 2004;44:749–57. 15572107 10.1016/j.neuron.2004.11.011

[98] Bosch M, Castro J, Saneyoshi T, Matsuno H, Sur M, Hayashi Y. Structural and molecular remodeling of dendritic spine substructures during long-term potentiation. Neuron. 2014;82:444–59. 24742465 10.1016/j.neuron.2014.03.021 PMC4281348

[99] De Rubeis S, Pasciuto E, Li KW, Fernandez E, Di Marino D, Buzzi A, et al. CYFIP1 coordinates mRNA translation and cytoskeleton remodeling to ensure proper dendritic spine formation. Neuron. 2013;79:1169–82. 24050404 10.1016/j.neuron.2013.06.039 PMC3781321

[100] Marcotrigiano J, Gingras AC, Sonenberg N, Burley SK. Cap-dependent translation initiation in eukaryotes is regulated by a molecular mimic of eIF4G. Mol Cell. 1999;3:707–16. 10394359 10.1016/s1097-2765(01)80003-4

[101] Mariano V, Kanellopoulos AK, Ricci C, Di Marino D, Borrie SC, Dupraz S, et al. Intellectual disability and behavioral deficits linked to CYFIP1 missense variants disrupting actin polymerization. Biol Psychiatry. 2024;95:161–74. 37704042 10.1016/j.biopsych.2023.08.027

[102] Hagerman RJ, Hagerman PJ. Fragile X syndrome: lessons learned and what new treatment avenues are on the horizon. Annu Rev Pharmacol Toxicol. 2022;62:365–81.34499526 10.1146/annurev-pharmtox-052120-090147

[103] Liu XS, Wu H, Krzisch M, Wu X, Graef J, Muffat J, et al. Rescue of Fragile X syndrome neurons by DNA methylation editing of the FMR1 gene. Cell. 2018;172:979–992.e976. 29456084 10.1016/j.cell.2018.01.012 PMC6375087

[104] Xie Y, Zhou B, Lin MY, Wang S, Foust KD, Sheng ZH. Endolysosomal deficits augment mitochondria pathology in spinal motor neurons of asymptomatic fALS mice. Neuron. 2015;87:355–70. 26182418 10.1016/j.neuron.2015.06.026 PMC4511489

[105] Gupta VK, Scheunemann L, Eisenberg T, Mertel S, Bhukel A, Koemans TS, et al. Restoring polyamines protects from age-induced memory impairment in an autophagy-dependent manner. Nat Neurosci. 2013;16:1453–60. 23995066 10.1038/nn.3512

[106] Komatsu M, Waguri S, Chiba T, Murata S, Iwata J, Tanida I, et al. Loss of autophagy in the central nervous system causes neurodegeneration in mice. Nature. 2006;441:880–4. 16625205 10.1038/nature04723

[107] Penzes P, Cahill ME, Jones KA, VanLeeuwen JE, Woolfrey KM. Dendritic spine pathology in neuropsychiatric disorders. Nat Neurosci. 2011;14:285–93. 21346746 10.1038/nn.2741 PMC3530413

[108] Zhao H, Zhao YG, Wang X, Xu L, Miao L, Feng D, et al. Mice deficient in Epg5 exhibit selective neuronal vulnerability to degeneration. J Cell Biol. 2013;200:731–41. 23479740 10.1083/jcb.201211014 PMC3601354

[109] Dong S, Aguirre-Hernandez C, Scrivo A, Eliscovich C, Arias E, Bravo-Cordero JJ, et al. Monitoring spatiotemporal changes in chaperone-mediated autophagy in vivo. Nat Commun. 2020;11:645. 32005807 10.1038/s41467-019-14164-4 PMC6994528

[110] Zhang Z, Yan J, Bowman AB, Bryan MR, Singh R, Aschner M. Dysregulation of TFEB contributes to manganese-induced autophagic failure and mitochondrial dysfunction in astrocytes. Autophagy. 2020;16:1506–23. 31690173 10.1080/15548627.2019.1688488 PMC7469609

[111] Kast DJ, Dominguez R. The cytoskeleton-autophagy connection. Curr Bio. 2017;27:R318–R326. 28441569 10.1016/j.cub.2017.02.061 PMC5444402

[112] Gkogkas CG, Khoutorsky A, Ran I, Rampakakis E, Nevarko T, Weatherill DB, et al. Autism-related deficits via dysregulated eIF4E-dependent translational control. Nature. 2013;493:371–7. 23172145 10.1038/nature11628 PMC4133997

[113] Zhang J, Abdel-Rahman AA. Nischarin as a functional imidazoline (I1) receptor. FEBS Lett. 2006;580:3070–4. 16678176 10.1016/j.febslet.2006.04.058

[114] Alahari SK. Nischarin inhibits Rac induced migration and invasion of epithelial cells by affecting signaling cascades involving PAK. Exp Cell Res. 2003;288:415–24. 12915132 10.1016/s0014-4827(03)00233-7

[115] Srdic-Rajic T, Nikolic K, Cavic M, Djokic I, Gemovic B, Perovic V, et al. Rilmenidine suppresses proliferation and promotes apoptosis via the mitochondrial pathway in human leukemic K562 cells. Eur J Pharm Sci. 2016;81:172–80. 26598394 10.1016/j.ejps.2015.10.017

[116] Reid JL. Rilmenidine: a clinical overview. Am J Hypertens. 2000;13:106S–111S. 10921529 10.1016/s0895-7061(00)00226-0

[117] Trimarco B, Rosiello G, Sarno D, Lorino G, Rubattu S, DeLuca N, et al. Effects of one-year treatment with rilmenidine on systemic hypertension-induced left ventricular hypertrophy in hypertensive patients. Am J Cardiol. 1994;74:36A–42A. 7998584 10.1016/0002-9149(94)90040-x

[118] Bauduceau B, Mayaudon H, Dupuy O. Rilmenidine in the hypertensive type-2 diabetic: a controlled pilot study versus captopril. J Cardiovasc Risk. 2000;7:57–61. 10785875 10.1177/204748730000700110

[119] Fauvel JP, Najem R, Ryon B, Ducher M, Laville M. Effects of rilmenidine on stress-induced peak blood pressure and renal function. J Cardiovasc Pharmacol. 1999;34:41–45. 10413065 10.1097/00005344-199907000-00007

[120] Vuu YM, Roberts CT, Rastegar M. MeCP2 is an epigenetic factor that links DNA methylation with brain metabolism. Int J Mol Sci. 2023;24:4218. 36835623 10.3390/ijms24044218 PMC9966807

[121] Prem S, Dev B, Peng C, Mehta M, Alibutud R, Connacher RJ, et al. Dysregulation of mTOR signaling mediates common neurite and migration defects in both idiopathic and 16p11.2 deletion autism neural precursor cells. eLife. 2024;13:e82809. 38525876 10.7554/eLife.82809 PMC11003747

[122] Zhou J, Blundell J, Ogawa S, Kwon CH, Zhang W, Sinton C, et al. Pharmacological inhibition of mTORC1 suppresses anatomical, cellular, and behavioral abnormalities in neural-specific Pten knock-out mice. J Neurosci. 2009;29:1773–83. 19211884 10.1523/JNEUROSCI.5685-08.2009 PMC3904448

