# Supplementary material for: Autophagy controls the hippocampal postsynaptic organization and affects cognition in a mouse model of Fragile X syndrome
Source: Mol Psychiatry. 2025 Sep 10;31(1):75–93. doi: 10.1038/s41380-025-03207-6 (PMC12700825; doi:10.1038/s41380-025-03207-6)

Supplemental Figure Legends

Suppl Figure 1. GO biological processes of overlapped proteins. The PANTHER Overrepresentation Test was performed on the dataset of 289 overlapped proteins using the GO biological processes with *Mus Musculus* genome database. The y-axis shows the top 30 GO biological processes, where the categories related to “Synapse”, “Dendrites”, and “Spines” are labeled with blue color.

Suppl Figure 2. Pharmacokinetic study after Rilmenidine injection. The concentrations of Rilmenidine were measured by LC-MS/MS in the plasma (**A**), and brain (**B**) at different time points (0, 0.5, 1.0, 2.0, and 4.0 hr) after a single injection (10 mg/kg, *i.p.*) (n = 4 mice in each time point).

Suppl Figure 3. Dose and time effects of Rilmenidine on autophagy in hippocampus. (**A**) WT and *Fmr1* KO mice were injected with Veh or Rilmenidine (Ril) at indicated doses (*i.p.*). Representative immunoblot of p62 in hippocampal lysates is shown. β-actin was used as loading control. Bar graph shows summary data (values were normalized to WT + Veh, n = 4 mice in each group). (**B**) WT and *Fmr1* KO mice were injected with Veh or Rilmenidine at 10 mg/kg (*i.p.*). Hippocampus tissues were collected at the indicated time points after injection. Protein lysates were probed for p62. β-actin was used as loading control. Bar graph shows summary data (values were normalized to WT + Veh at 0 hr, n = 4 mice for each time point). Values are shown as mean ± s.e.m. Significance was calculated by ANOVA followed by Tukey’s test. $^{**}p < 0.01$, *N S*: no significant difference. Each circle represents data from an individual mouse.

Suppl Figure 4. Effect of Rilmenidine on bodyweight and growth of mice. WT and *Fmr1* KO mice were injected with Veh or Rilmenidine at 10 mg/kg (*i.p.*) daily for one week. Body weight (**A**) and body length (**B**) were recorded. Values are shown as mean ± s.e.m. Significance was calculated by ANOVA followed by a Tukey’s test. Each circle represents data from an individual mouse (n = 10 mice in each group). Mice used are 5-week-old.

Suppl Figure 5. Effect of Rilmenidine on wild type mice. Wild-type (WT) mice were injected daily with vehicle (saline) or Rilmenidine (*i.p.,* 10mg/kg BW) (WT + Ril) daily for 1 week. (**A**) Brain frozen sections

were subjected to immunostaining of p62 together with NeuN to mark neurons. Scale bar, 25 µm. (**B**) Summary bar graph shows the normalized fluorescent intensity of p62 puncta in A. (**C**) Hippocampal lysates from treated mice were assessed with Western blot for p62. (**D**) Bar graph shows summary of normalized data of C. (**E**) Mouse brains were subjected to Golgi staining and all spines located on apical dendrites on CA1 pyramidal neurons were analyzed. Scale bar, 3 µm. (**F**) Spine number per 10 µm of dendrite. (**G**) Analysis of stubby/mushroom and filopodial spine fractions. Significance was calculated by the t-test (unpaired, two-tailed). β-actin was used as a loading control. Values reflect mean ± s.e.m. Each circle represents data from an individual mouse in B, D F and G (n = 4 in each group). Mice used are 5-week-old.

Suppl Figure 6. Time spent exploring novel and familiar objects by Rilmenidine treated mice. WT and *Fmr1* KO mice were treated with Vehicle or Rilmenidine (*i.p.,* 10mg/kg BW, daily for one week) (n = 9 mice in WT, n = 10 in *Fmr1* KO, and n = 10 in *Fmr1* KO + Ril). Significance was calculated by the t-test (unpaired, two-tailed). * $p < 0.05$. ** $p < 0.01$. *N S*: Values reflect mean ± s.e.m. Each circle represents data from an individual mouse. Mice are 5-week-old.

Suppl Figure 7. Additional behavioral tests of mice with autophagy activation. Wild-type (WT) and *Fmr1* KO mice were injected daily with vehicle (saline) or Rilmenidine (*i.p.,* 10mg/kg BW) (*Fmr1* KO + Ril) for 1 week as described in Fig.2. Then mice were subjected to: (**A**, **B**) Nest building test. (**A**) Nests after 24 hr. (**B**) Nest building scored on a scale of 1–5; (**C**, **D**) Open field test. (**C**) Representative track plots of mouse movement. (**D**) Bar graph shows times spent in central zone; and (**E**) Self-grooming test. Significance was calculated by one-way ANOVA followed by a Tukey's test. ** $p < 0.01$. Values reflect mean ± s.e.m. Each circle represents data from an individual mouse in B, D and E (n = 10). Mice used are 5-week-old.

Suppl Figure 8. Cognitive tests of mice with ATG7 knockdown in hippocampal neurons. (**A, B**) WT ($Atg7^{w/w}$), heterozygous $Atg7^{w/f}$, and homozygous $Atg7^{f/f}$ mice were bilaterally injected with AAV expressing Syn-Cre-GFP to hippocampus at P28 and then subjected to histology and behavioral tests at P35. (**C**) Brain frozen sections were immunostained with GFP together with NeuN to show the injection sites. Scale bar, 100 µm. (**D**) Protein lysates of hippocampus were assessed with Western blot for ATG7 and p62. (**E, F**)

Bar graphs show summary data (values were normalized to WT) of D (n = 4 mice in each group). (**G-I**) Visual memory was assessed by the novel object recognition task: (**G**) Representative heatmaps of mouse movement; (**H**) Time spent exploring novel and familiar objects (n = 8 mice in each group); (**I**) Preference index to the novel object. (**J**) Percentages of freezing response in familiar and novel contexts during the contextual fear conditioning test (n = 8 mice in each group). Significance was calculated by the t-test (unpaired, two-tailed) and one-way ANOVA followed by a Tukey's test. * $p < 0.05$, ** $p < 0.01$. Values reflect mean ± s.e.m. Each circle in E, F, H, I and J represents data from an individual mouse.

Suppl Figure 9. Validation of *Atg7* knockout in mouse brain. WT control mice (WT: $Cre^{-/-}$: $Atg7^{f/f}$), *Fmr1* KO control mice ($Fmr1^{-/y}$: $Cre^{-/-}$: $Atg7^{f/f}$), and *Fmr1* KO mice with neuron-specific *Atg7* knockout ($Fmr1^{-/y}$: $Cre^{+/-}$: $Atg7^{f/f}$) were injected (*i.p.*) with vehicle (Veh) or Rilmenidine (Ril) daily for one week. Lysates of collected hippocampus were assessed with Western blot for ATG7. β-actin was used as loading control. (**A**) Representative blots; (**B**) Bar graph shows summary data (values were normalized to WT control + Veh group, n = 4 mice in each group). Significance was calculated by ANOVA followed by a Tukey's test. * $p < 0.05$. Values reflect mean ± s.e.m. Each circle in B represents data from an individual mouse. Mice used are 5-week-old.

Suppl Figure 10. Effects of autophagy activation on PSD-95 and eIF4G1 in cultured neurons. (**A**) Primary neurons were cultured from the hippocampus of WT and *Fmr1* KO mice and treated with Veh (DMSO) or Rilmenidine (10 µM for 6 hr). Protein lysates were assessed with the Western blot of PSD-95 and eIF4G1. (**B**) Bar graphs show summary data. Significance was calculated by ANOVA followed by Tukey's test. * $p < 0.05$, ** $p < 0.01$. β-actin was used as a loading control. Values were normalized to WT with Veh and reflect mean ± s.e.m. Each circle represents data from an independent culture (n = 4).

Suppl Figure 11. Degradation of PSD-95 and eIF4G1 by autophagy. Primary neurons were cultured from the hippocampus of WT and *Fmr1* KO mice and treated with labeled drugs: WT (WT neurons with DMSO); *Fmr1* KO (*Fmr1* KO neurons with DMSO); *Fmr1* KO + Ril (*Fmr1* KO neurons with Rilmenidine, 10 µM for 6 hr) and WT + lyso Inhi (WT neurons with lysosomal inhibitors: 10 mM NH_4Cl with 50 µM leupeptin

for 6 hr). Lysates were extracted and immunoprecipitated with an antibody to ubiquitin. Whole-cell lysates (Input) and immunoprecipitants (IP) were immunoblotted (IB) for PSD-95 and eIF4G1. (**A, B**): Summary data for PSD-95 and eIF4G1 in input. Summary data were normalized to WT + DMSO. Significance was calculated by ANOVA followed by Tukey's test. Values reflect mean ± s.e.m. ** $p < 0.01$. Each circle represents data from an independent culture (n = 4).

Suppl Figure 12. Effects of Rilmenidine on PSD-95 and eIF4G1 in neuro-specific ATG7 KO mice. (**A**) WT: Cre$^{-/-}$: *Atg7*$^{f/f}$ mice, *Fmr1*$^{-/y}$: Cre$^{-/-}$: *Atg7*$^{f/f}$ mice, and *Fmr1*$^{-/y}$: Cre$^{+/-}$: *Atg7*$^{f/f}$ mice (neuron-specific *Atg7* knockout) were injected with Veh or Rilmenidine (*i.p.,* 10mg/kg BW). Hippocampal protein lysates were assessed with Western blot for PSD-95 and eIF4G1. (**B, C**) Bar graphs show summary data. n = 4 mice in each group. Significance was calculated by ANOVA followed by Tukey's test. ** $p < 0.01$. β-actin was used as a loading control. Values were normalized to WT with Veh and reflect mean ± s.e.m. Each circle represents data from an individual mouse (5-week-old).

Suppl Figure 13. Effects of Rilmenidine on mRNAs of PSD-95 *(Dlg4)* and *Eif4g1*. 5-week-old WT mice were injected with Veh or Rilmenidine at 10 mg/kg (*i.p.*). 4 hr after injection, hippocampus tissues were collected and analyzed with real time RT-PCR to examine mRNA levels of PSD-95 *(Dlg4)* and *Eif4g1.* (**A**) Amplification plot; (**B**) Bar graphs show summary data (values were normalized to Veh). Values are shown as mean ± s.e.m. n = 4 mice in each group. Significance was calculated by the t-test (unpaired, two-tailed). *N S.*: no significant difference. Each circle in B represents data from an individual mouse.

Suppl Figure 14. Effect of autophagy activation on PSD-95 levels in dendrites. (**A**) Primary hippocampal neurons were cultured from WT and *Fmr1* KO mice, transfected with lentivirus expressing Syn-RFP to show dendrites, and treated with Veh or Ril (Rilmenidine, 10 μM for 6 hr). Images show immunolabeling of PSD-95 in dendritic area. Scale bar, 3 μm. (**B**) Summary bar graph shows fluorescent intensity of PSD-95 puncta. n = 4 cultures in each group. Significance was calculated by ANOVA followed by a Tukey's test. ** $p < 0.01$. Values were normalized to WT with Veh and reflect mean ± s.e.m. Each circle in B represents data from an independent culture (n = 4).

Suppl Figure 15. Association of eIF4E with CYFIP1 in neurons with eIF4G1 knockdown. (**A**) Primary hippocampal neurons were cultured from WT mice and transfected with lentivirus expressing eIF4G1 shRNA or control shRNA. (**A**) 5 days after transfection, protein lysates of neurons were immunoprecipitated with an antibody to eIF4E. Lysates (Input) and immunoprecipitants (IP) were immunoblotted (IB) for eIF4E, eIF4G1, and CYFIP1. (**B**-**F**) Bar graphs show summary data of A. Significance was calculated by the t-test (unpaired, two-tailed). β-actin was used as a loading control. ** $p < 0.01$. Values reflect mean ± s.e.m. Each circle represents data from an individual culture in B-F (n = 4 in each group).

Suppl Figure 16. Protein levels of eIF4G1 and CYFIP1 in lysates of hippocampus. WT and *Fmr1* KO mice were injected with vehicle (as WT and *Fmr1* KO groups) or Rilmenidine (*Fmr1* KO + Ril). Protein lysates of hippocampal tissues were immunoprecipitated with an antibody to eIF4E. Lysates (Input) and immunoprecipitants (IP) were immunoblotted (IB) for eIF4E, eIF4G1, and CYFIP1. A naïve IgG antibody was used as negative control. (**A, B**) Bar graphs show summary data of eIF4G1 and CYFIP1 in input. Significance was calculated by ANOVA followed by a Tukey's test. * $p < 0.05$, ** $p < 0.01$. β-actin was used as a loading control. Values in bar graphs were normalized to WT and reflect mean ± s.e.m. Each circle represents data from an individual mouse (5-week-old, n = 4 in each group).

Suppl Figure 17. Effect of autophagy activation on phosphorylation of Cofilin1. (**A**) 5-week-old WT mice were injected with Veh or Rilmenidine at 10 mg/kg (i.p.). 4 hr after injection, hippocampal synaptosomes were isolated and protein lysates were immunoblotted with total Cofilin1 and p-Cofilin1 (Ser-3). (**B**) Bar graph shows ratio of p-Cofilin1/total Cofilin1. Significance was calculated by ANOVA followed by a Tukey's test. ** $p < 0.01$. *** $p < 0.001$. β-actin was used as a loading control. Values in bar graphs were normalized to WT and reflect mean ± s.e.m. Each circle represents data from an individual mouse (5-week-old, n = 4 mice in each group).

Suppl Figure 18. Effects of central Rilmenidine infusion on cognition and actin dynamics of *Fmr1* KO mice. (**A**) WT and *Fmr1* KO mice were implanted with cannulas bilaterally to lateral ventricles and (**B**)

infused with aCSF (as WT and *Fmr1* KO), or Rilmenidine (as *Fmr1* KO +Ril) daily for one week. (**C**) Protein lysates of hippocampus were assessed with Western blot for p62, PSD-95 and eIF4G1. (**D, E, F**) Bar graphs show summary data (values were normalized to WT). n = 4 in each group. (**G**) Percentages of freezing response in familiar and novel contexts during the contextual fear conditioning test (n = 9 in WT, n = 8 in *Fmr1* KO, and n = 8 in *Fmr1* KO + Ril). (**H**, **I**) Visual memory was assessed by the novel object recognition task: (**H**) Representative heatmaps of mouse movement; (**I**) Time spent exploring novel and familiar objects (n = 9 mice in each group). (**J**) Hippocampal synaptosomes were isolated and protein lysates were assessed with F/G-actin ratio by Western blot. (**K**) Bar graph shows summarized F/G ratio (values were normalized to WT, n = 4 mice in each group). Significance was calculated by the t-test (unpaired, two-tailed) and ANOVA followed by a Tukey's test. * $p < 0.05$, ** $p < 0.01$, *** $p < 0.001$. Values reflect mean ± s.e.m. Each circle in D-G, **I**, and K represents data from an individual mouse. All mice used are 5-week-old.

Suppl Figure 19. Activation of autophagy reduced Cofilin1 phosphorylation in human FXS neurons. Neurons were differentiated from unaffected (control) and FXS human iPSCs and treated with Veh or Rilmenidine (Ril). Protein lysates were assessed with Western blot for p-Cofilin1 (S-3) and total Cofilin1. (**A**) Representative blots. (**B**) Bar graph shows summary data (values were normalized to control + Veh). Significance was calculated by ANOVA followed by a Tukey's test. ** $p < 0.01$. Values reflect mean ± s.e.m. Each circle in b represents data from an individual culture (n = 4 culture in each group).

Suppl Figure 20. Activation of autophagy corrected the aberrant actin assembly in human FXS neurons. (**A**) Information of human iPSCs. (**B**) Neurons were differentiated from unaffected (control) and FXS iPSCs. FMRP expression was examined by immunostaining of FMRP and neuronal marker Tuj-1. Scale bar, 15 μm. (**C**) iPSCs-derived neurons were treated with DMSO as vehicle or Rilmenidine (Ril) (10μM for 6 hr), and immunostained with p62 together with Tuj-1. Scale bar, 15 μm. (**D**) Summary bar graph shows fluorescent intensity of p62 puncta in C (normalized to the value of control). (**E, F**) iPSCs-derived neurons were treated with DMSO as vehicle or Rilmenidine (Ril) (10μM for 6 hr), and immunostained with eIF4G1/PSD-95 together with Tuj-1. Scale bar, 20 μm. (**G**, **H**) Summary bar graphs show fluorescent intensities of eIF4G1 and PSD-95 puncta. (**I**) iPSCs derived neurons were treated with Veh or Rilmenidine

(10 μM) for 6 hr and imaged with F-actin. Scale bar, 3 μm. (**J**) Summary bar graph shows fluorescent intensity of F-actin. Significance was calculated by one-way ANOVA followed by a Tukey's test. ** $p < 0.01$. Values reflect mean ± s.e.m. Each circle in D, G, H, and J represents data from an individual experiment.

Suppl Figure 21. Effect of Rilmendine on protein synthesis in *Fmr1* KO hippocampal neurons. Primary neurons were cultured from the hippocampus of *Fmr1* KO mice and treated with Veh (DMSO) or Rilmenidine (10 μM for 6 hr). Protein synthesis was measured with SUnSET. (**A**) Representative Western blot showing the levels of puromycin incorporation. GAPDH was used as loading control; (**B**) quantification of puromycin normalized to GAPDH. Significance was calculated by the t-test (unpaired, two-tailed). * $p < 0.05$. Values reflect mean ± s.e.m. Each circle represents data from an independent culture (n = 4).

Supplemental Figure 1

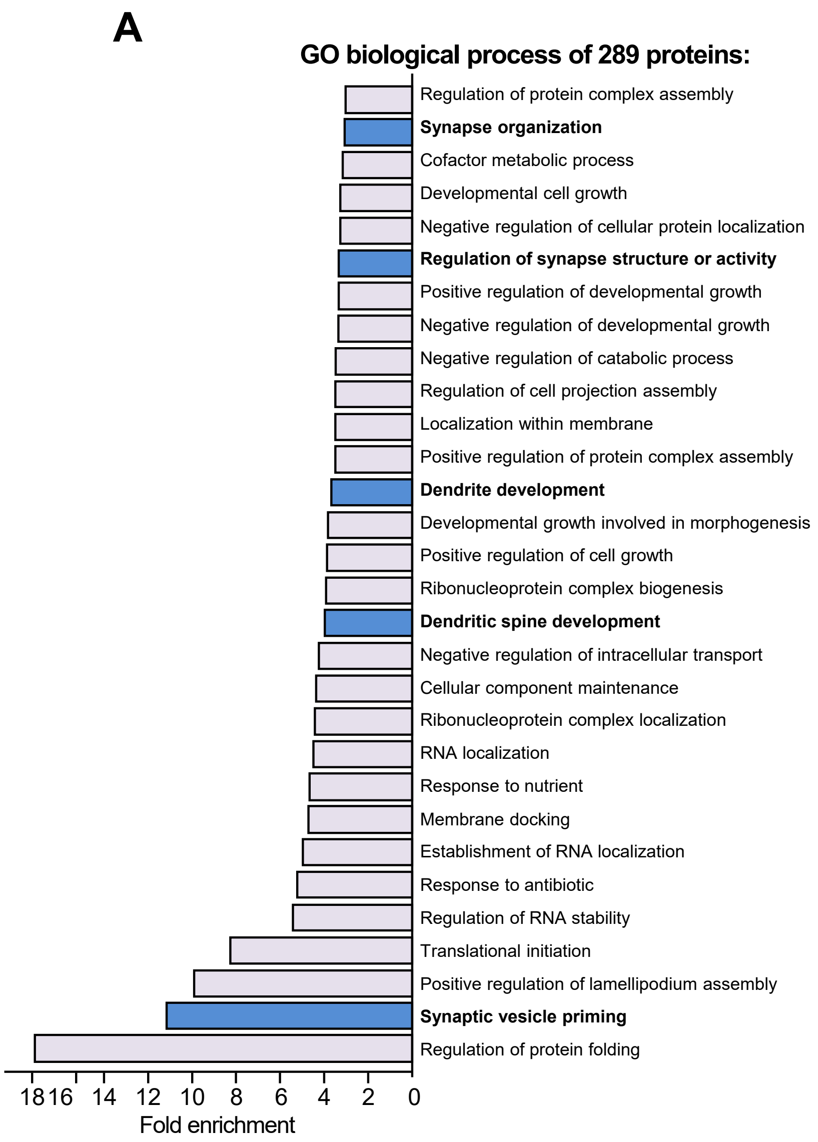

Supplemental Figure 2

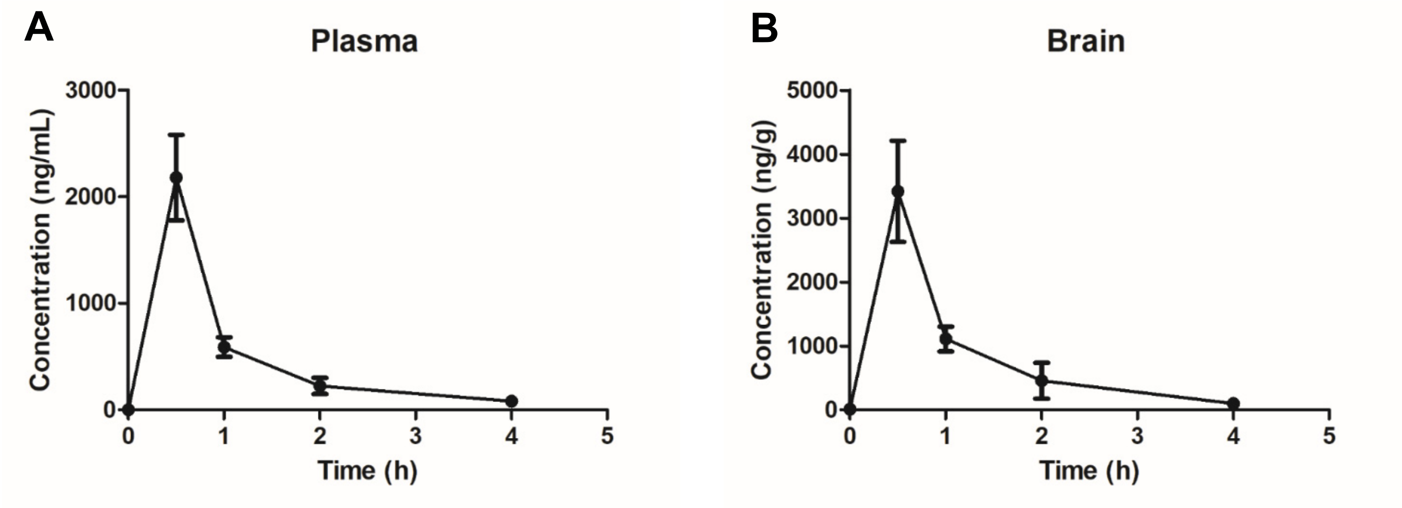

Supplemental Figure 3

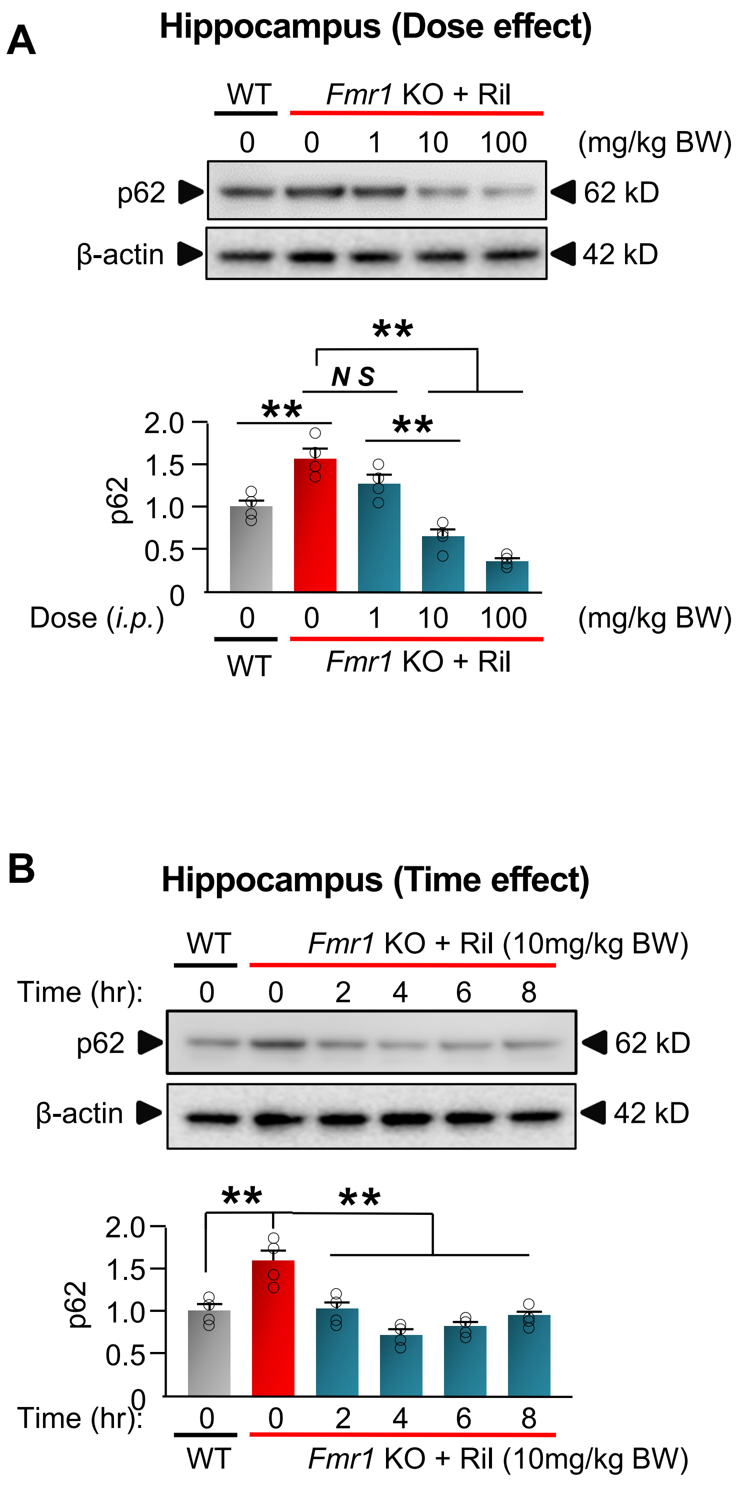

Supplemental Figure 4

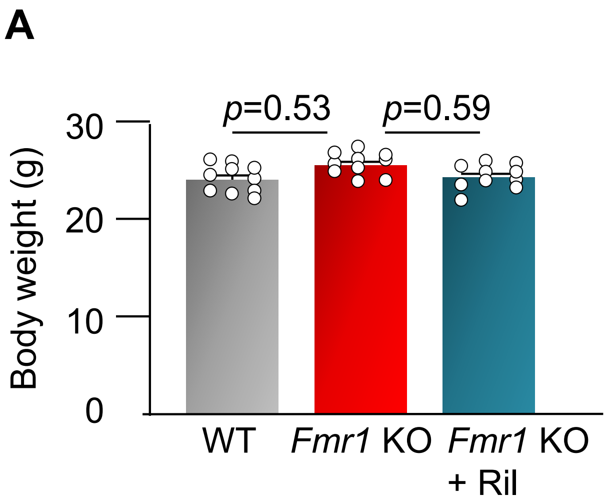

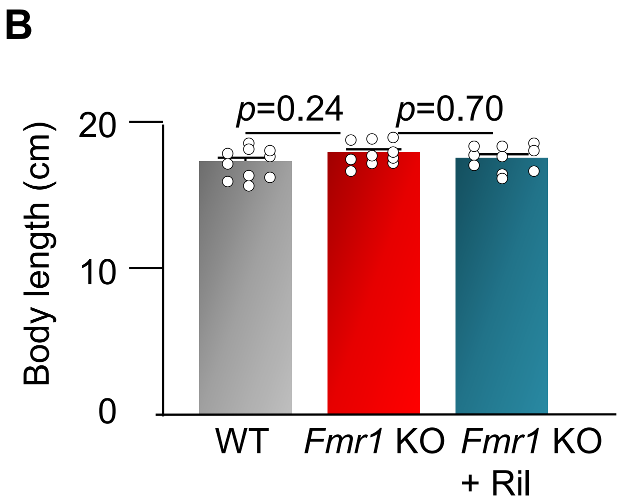

Supplemental Figure 5

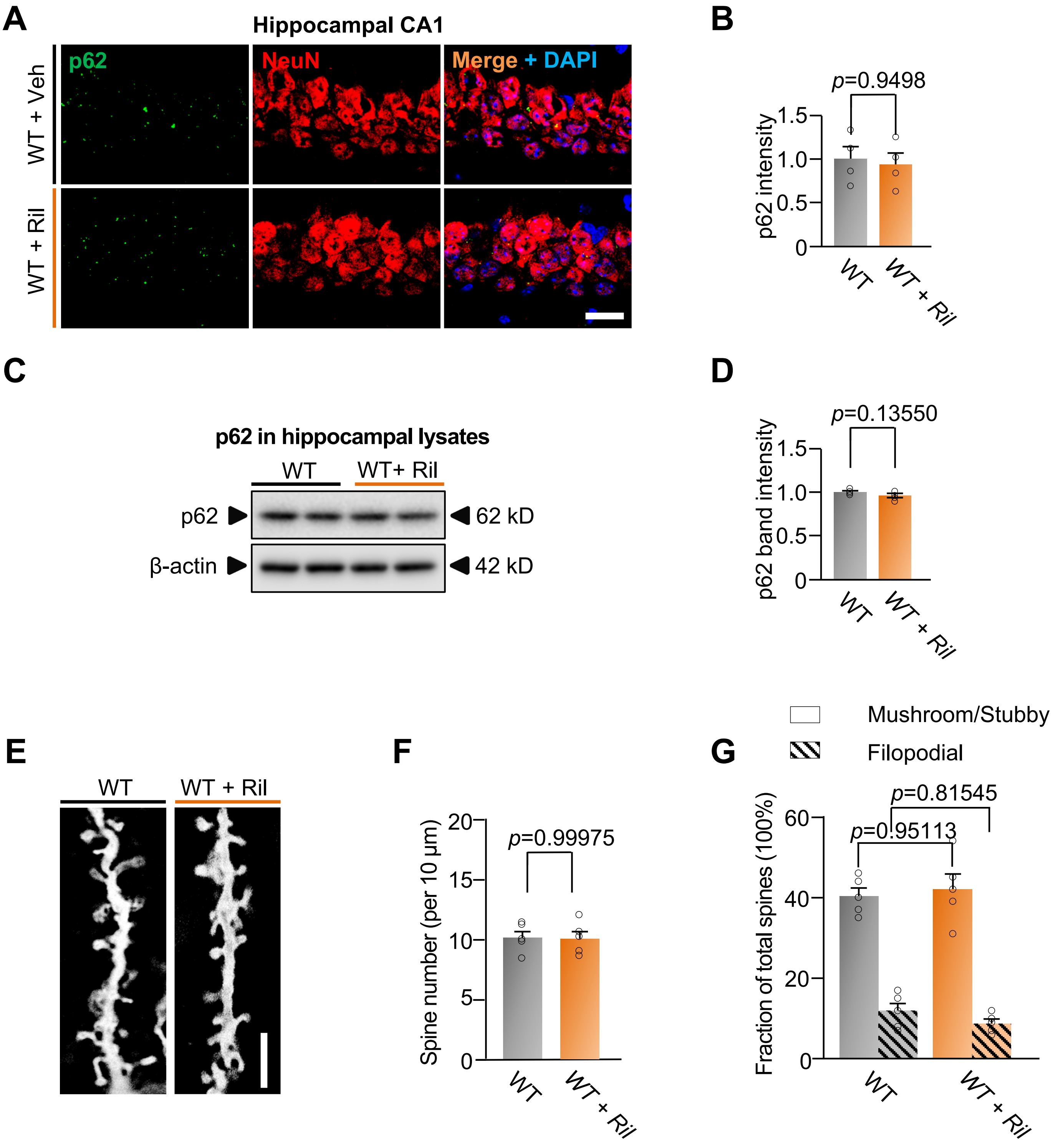

Supplemental Figure 6

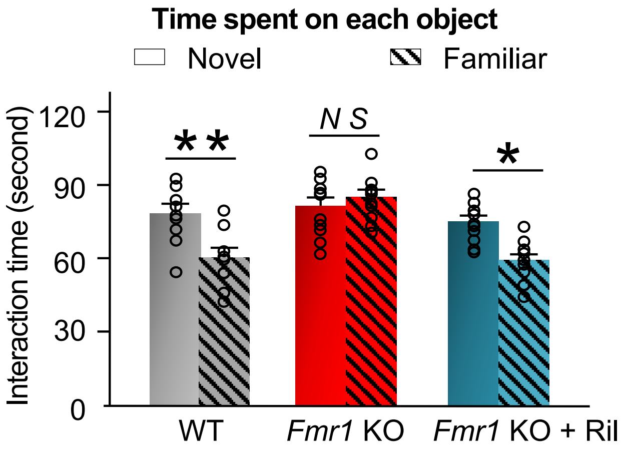

Supplemental Figure 7

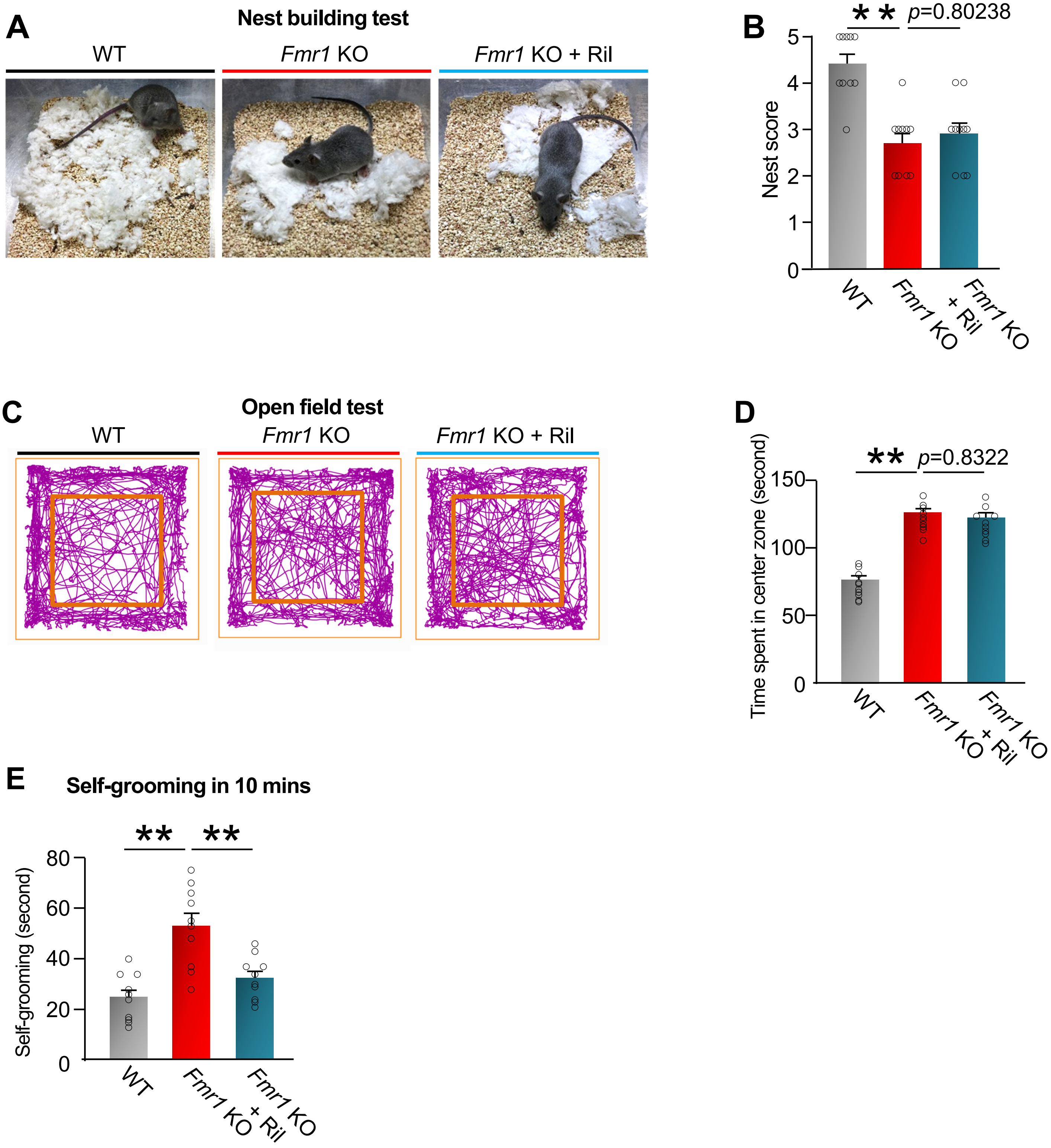

Supplemental Figure 8

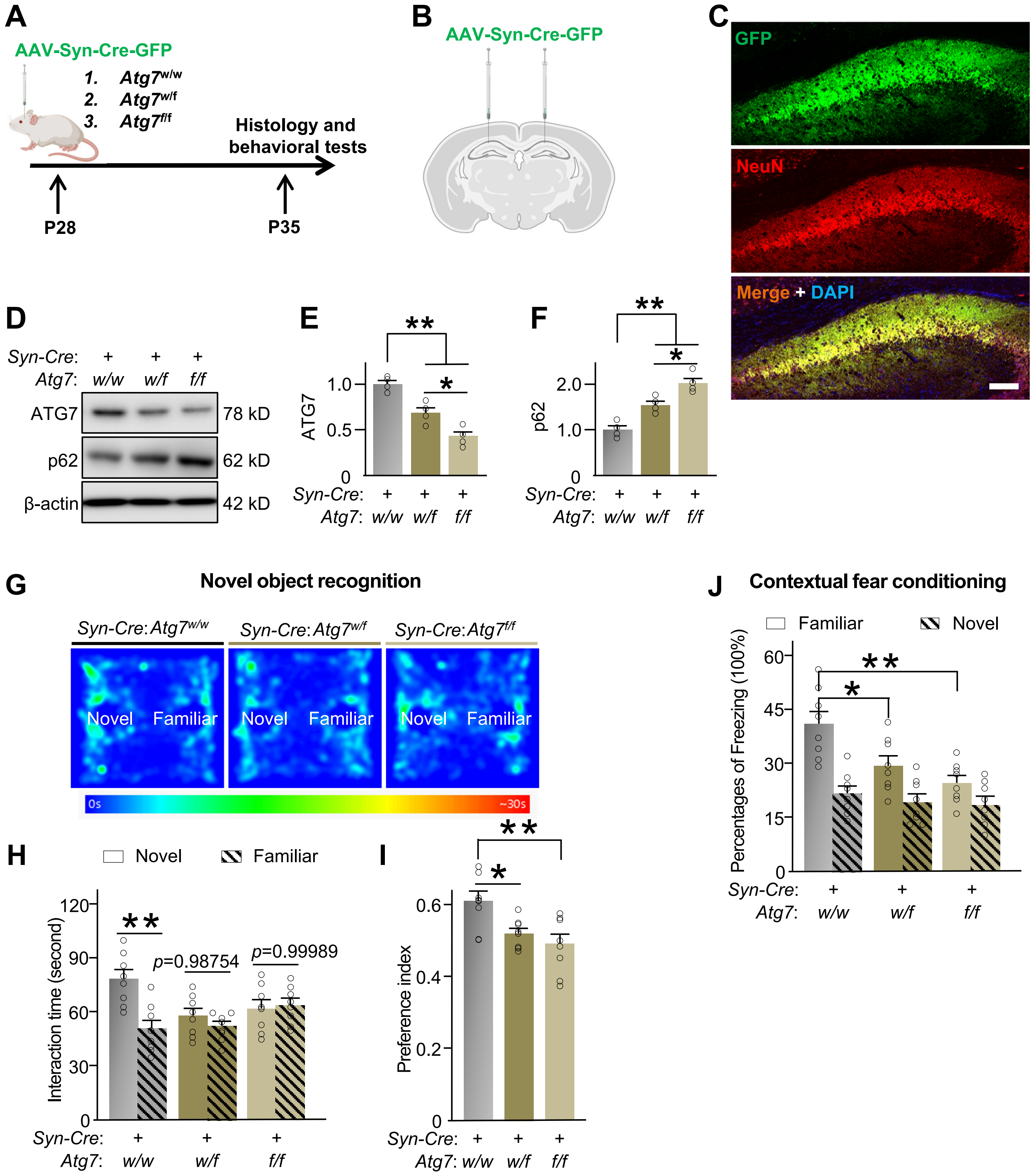

Supplemental Figure 9

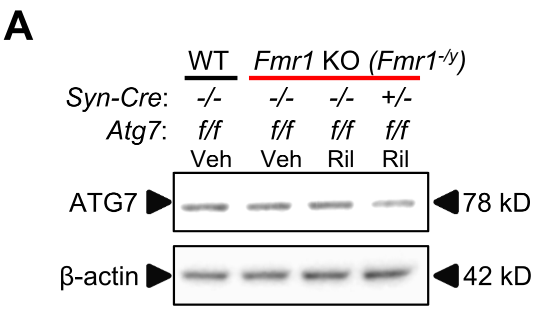

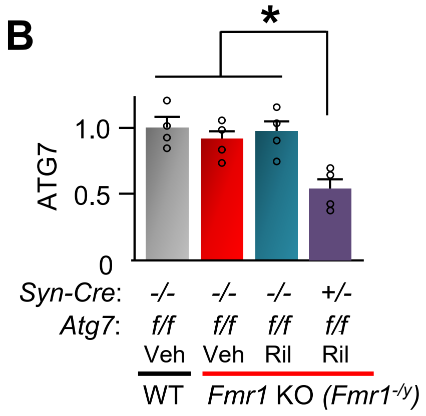

Supplemental Figure 10

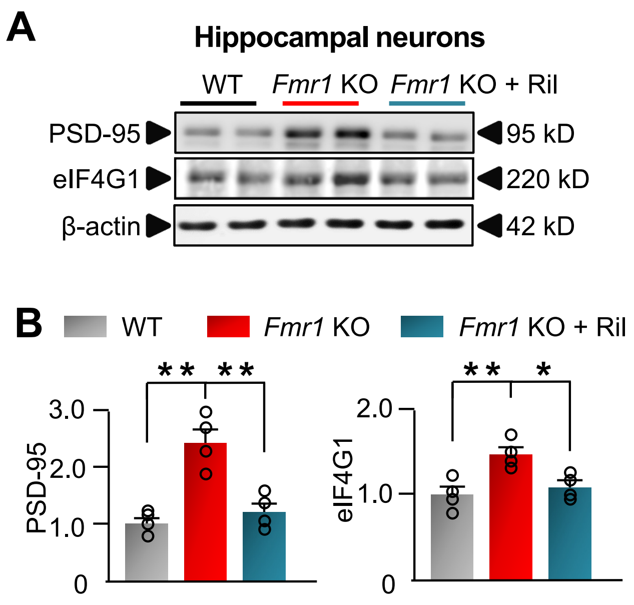

Supplemental Figure 11

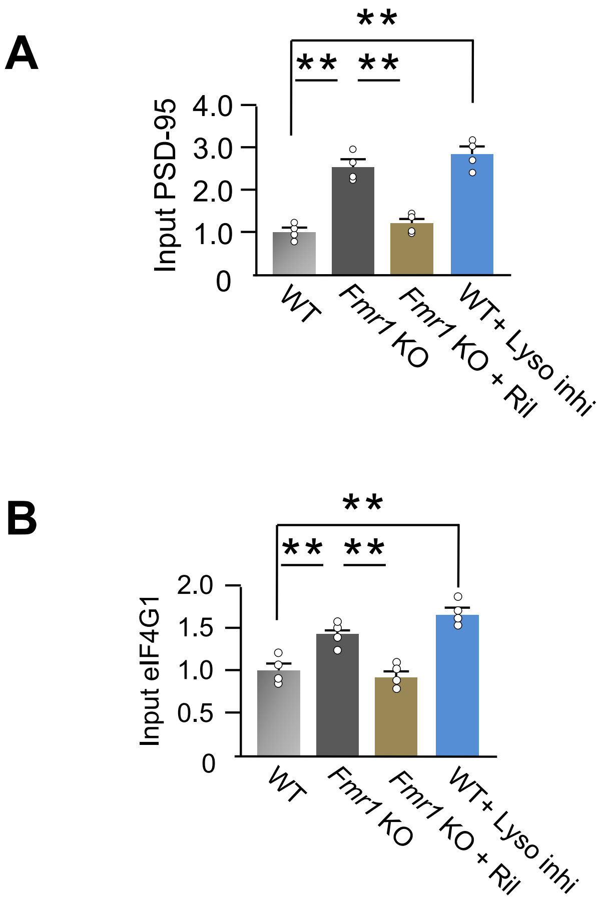

Supplemental Figure 12

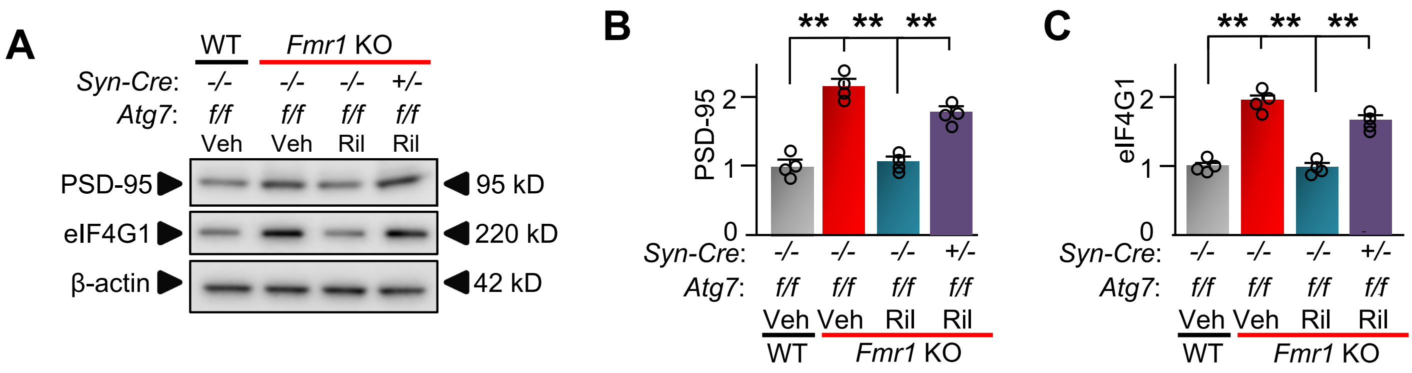

Supplemental Figure 13

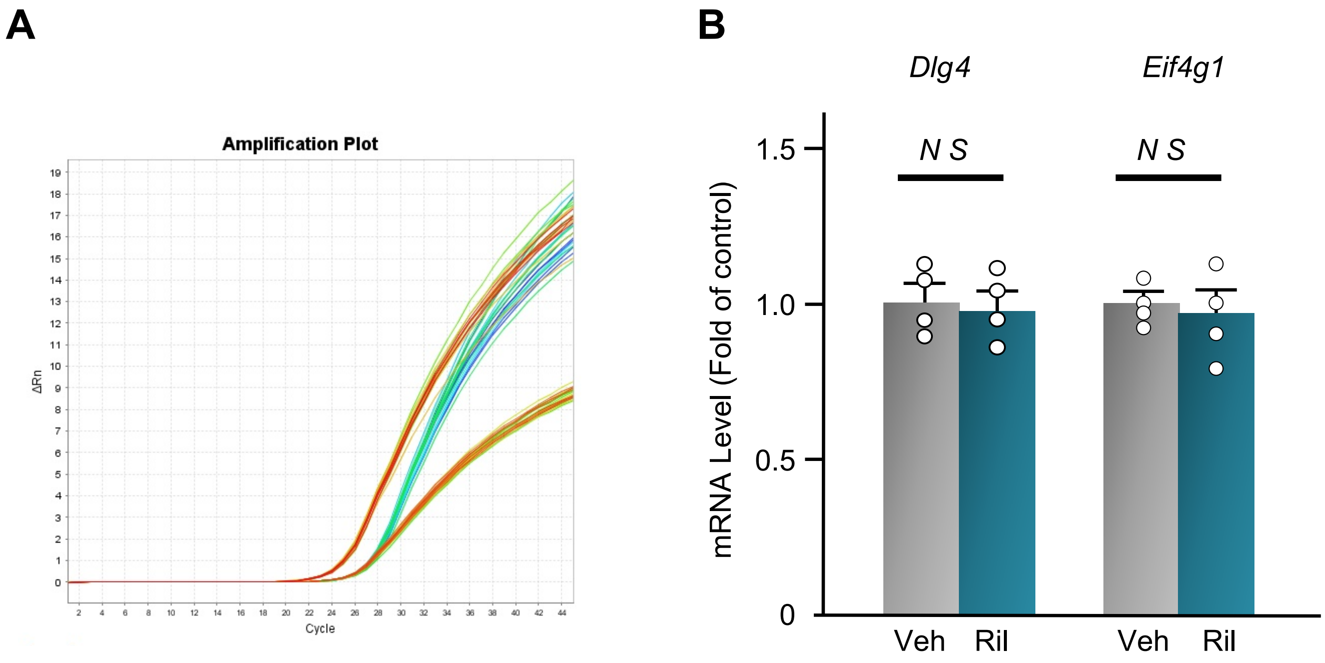

Supplemental Figure 14

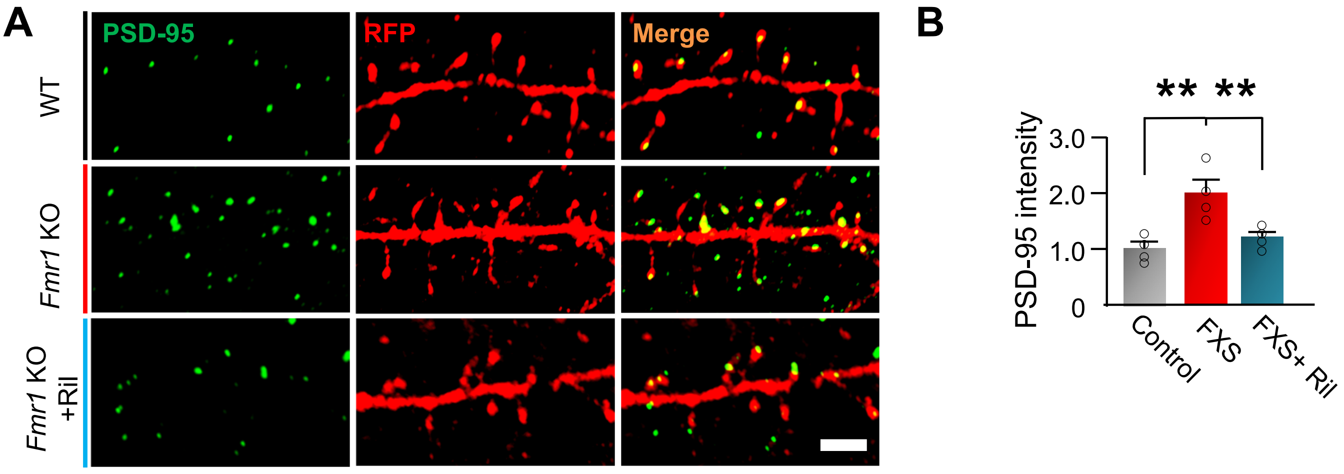

Supplemental Figure 15

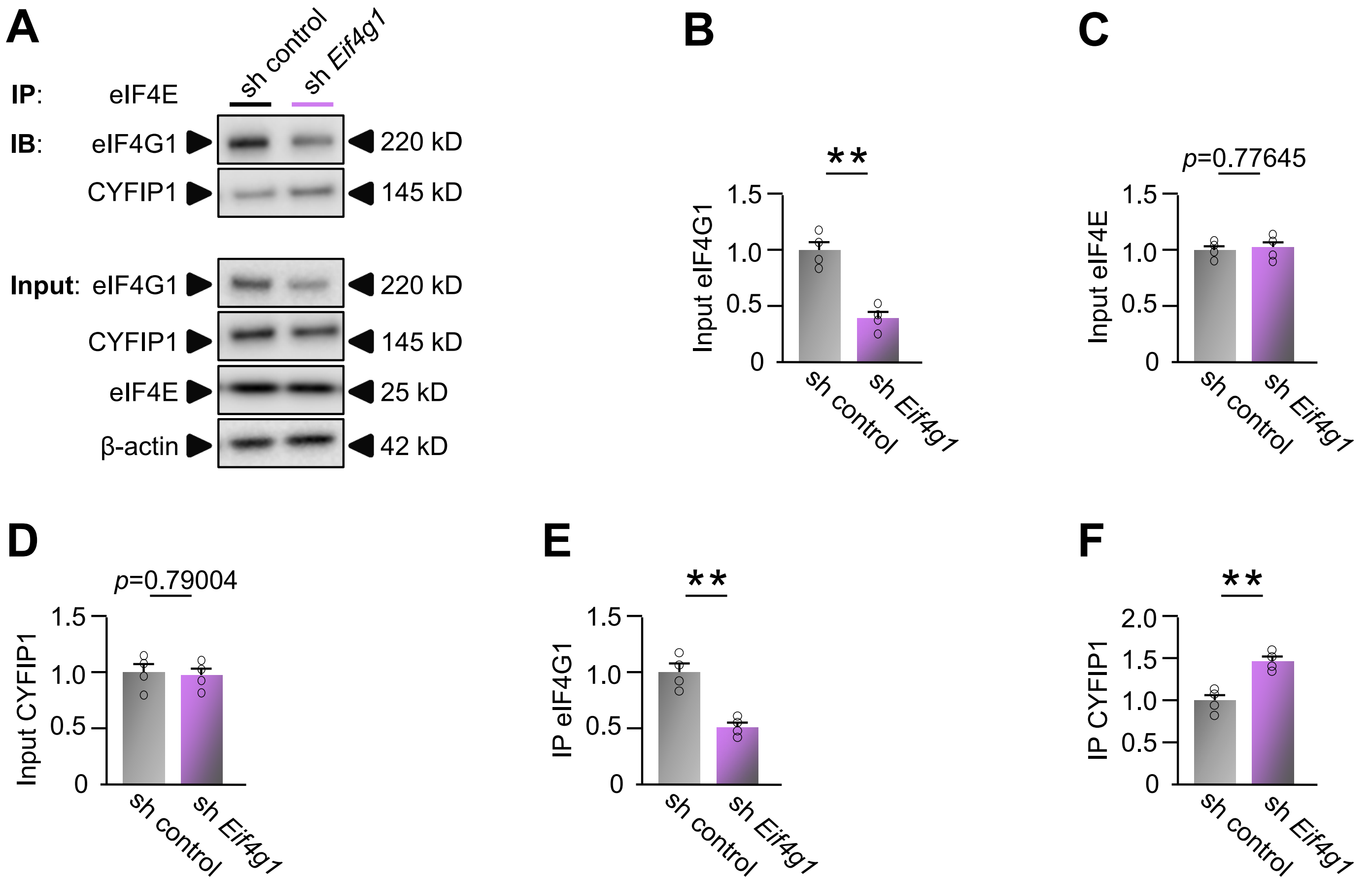

Supplemental Figure 16

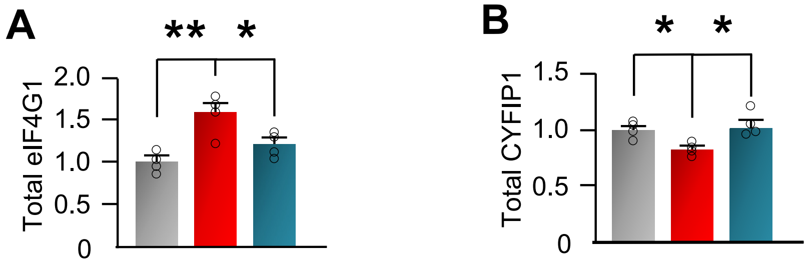

Supplemental Figure 17

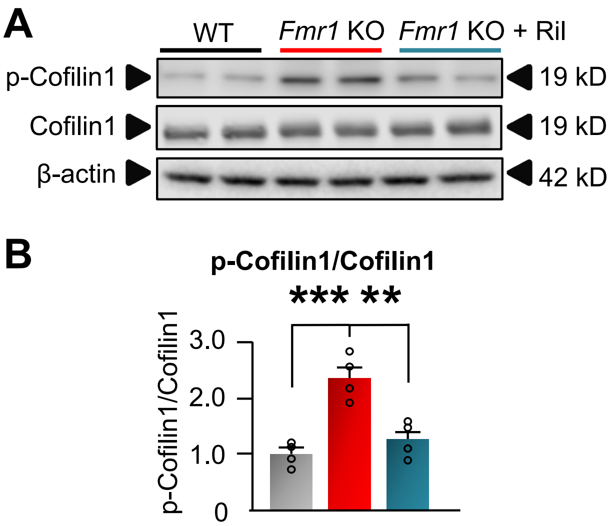

Supplemental Figure 18

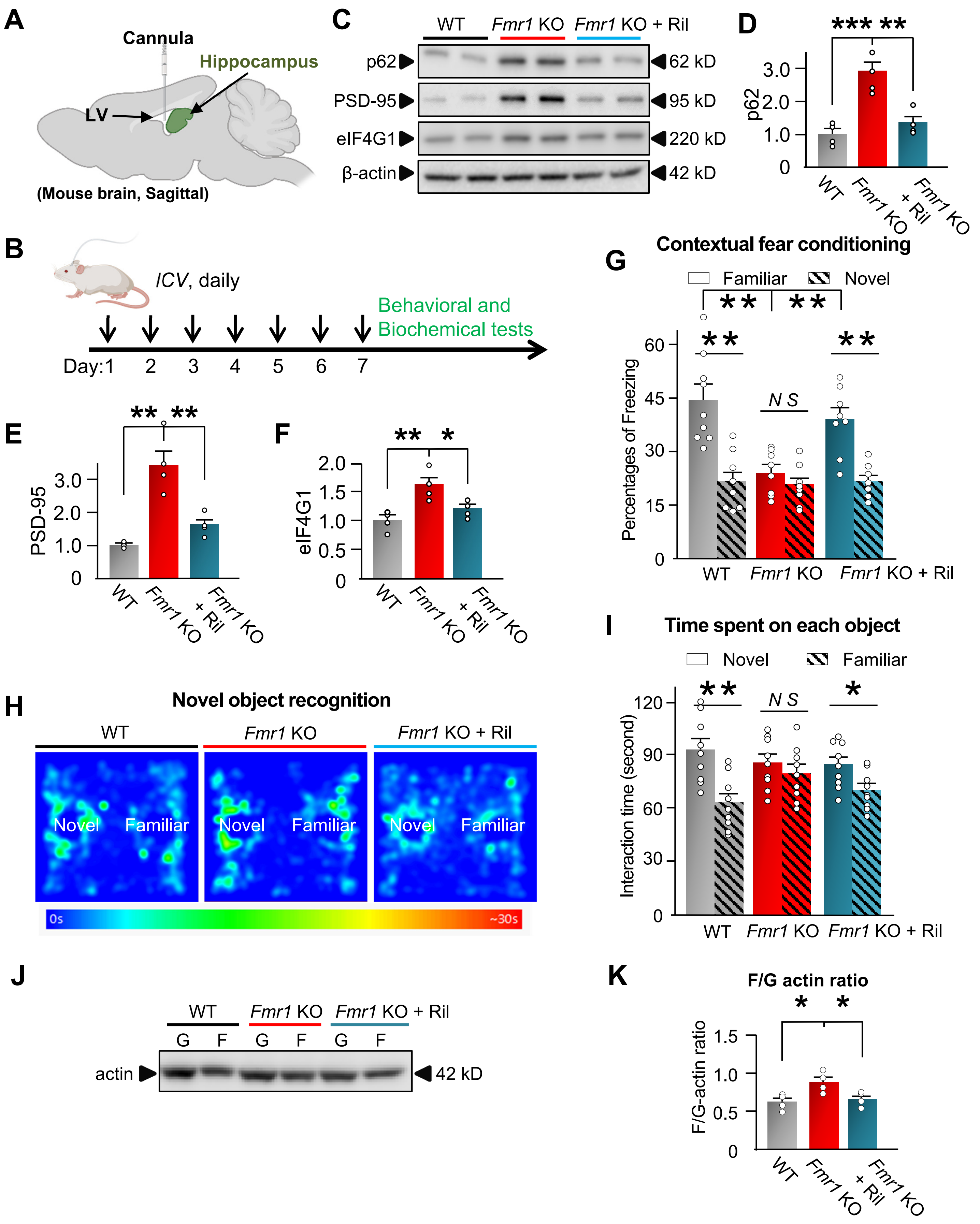

Supplemental Figure 19

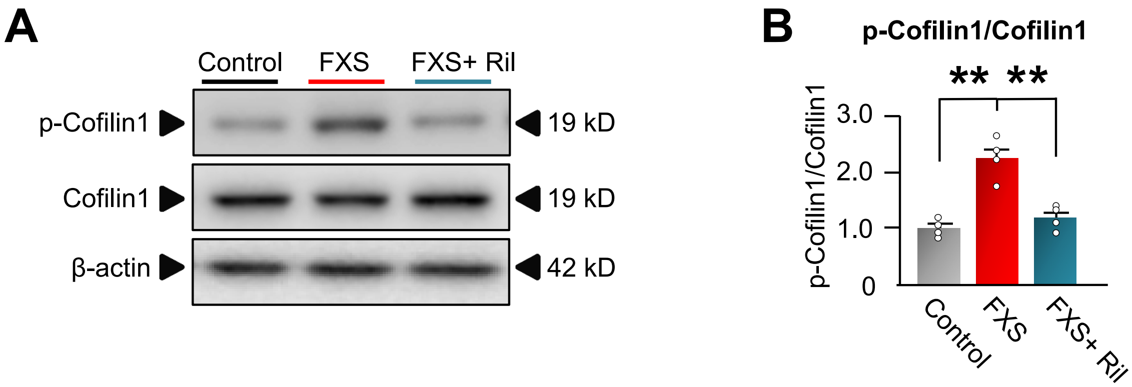

Supplemental Figure 20

A

Information of human iPSCs

iPSCs	Diagnosis of patient	Gender of patient	CGG Repeat
Control (**C603**)	unaffected	Male	31
FXS (**FX08-23**)	FXS and Intellectual disability	Male	>435

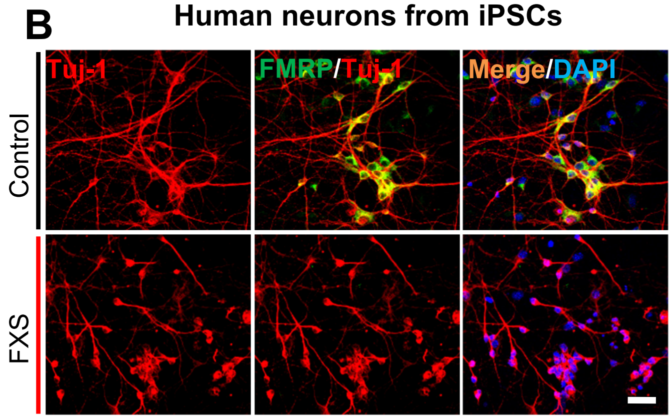

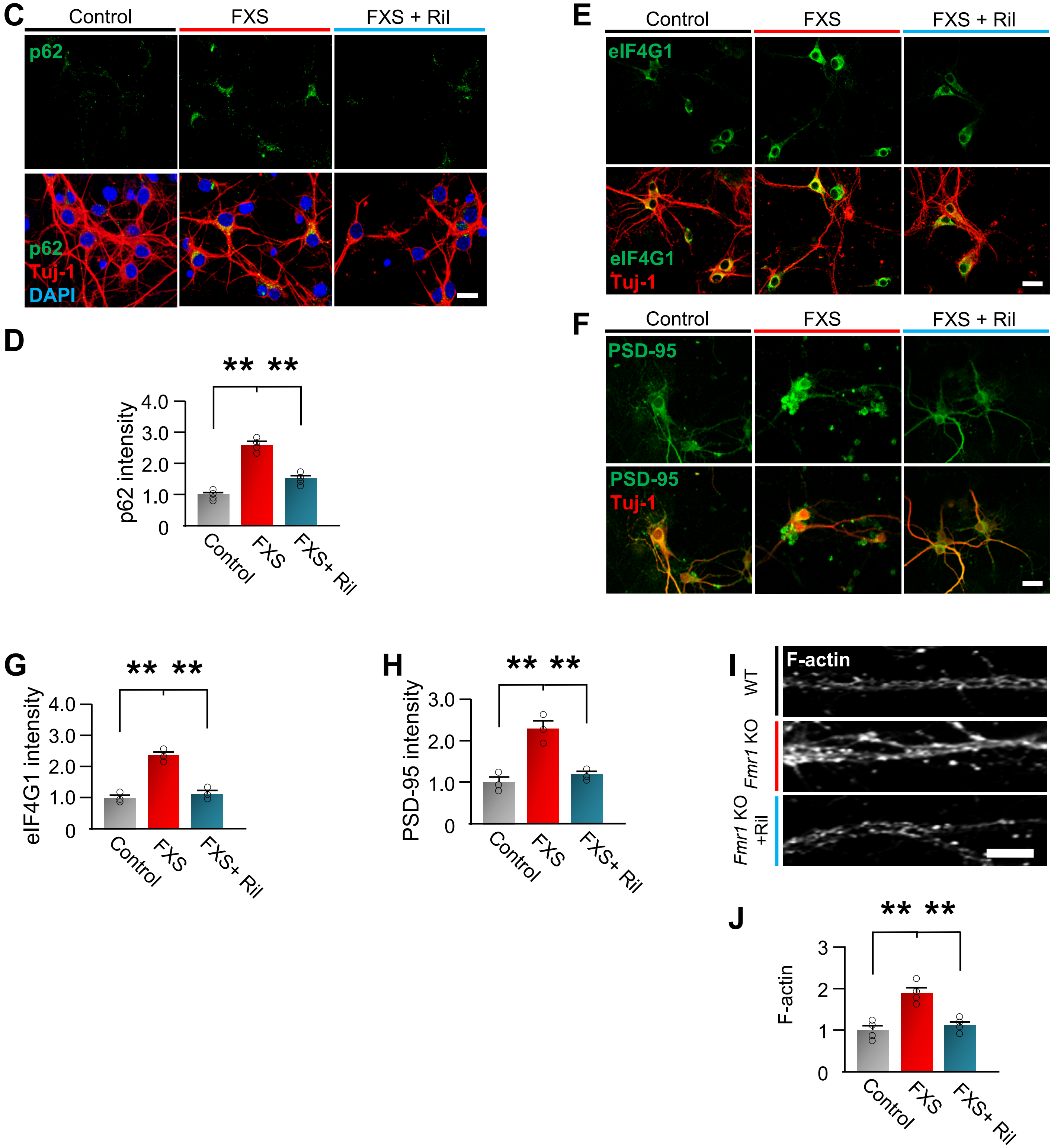

Supplemental Figure 21

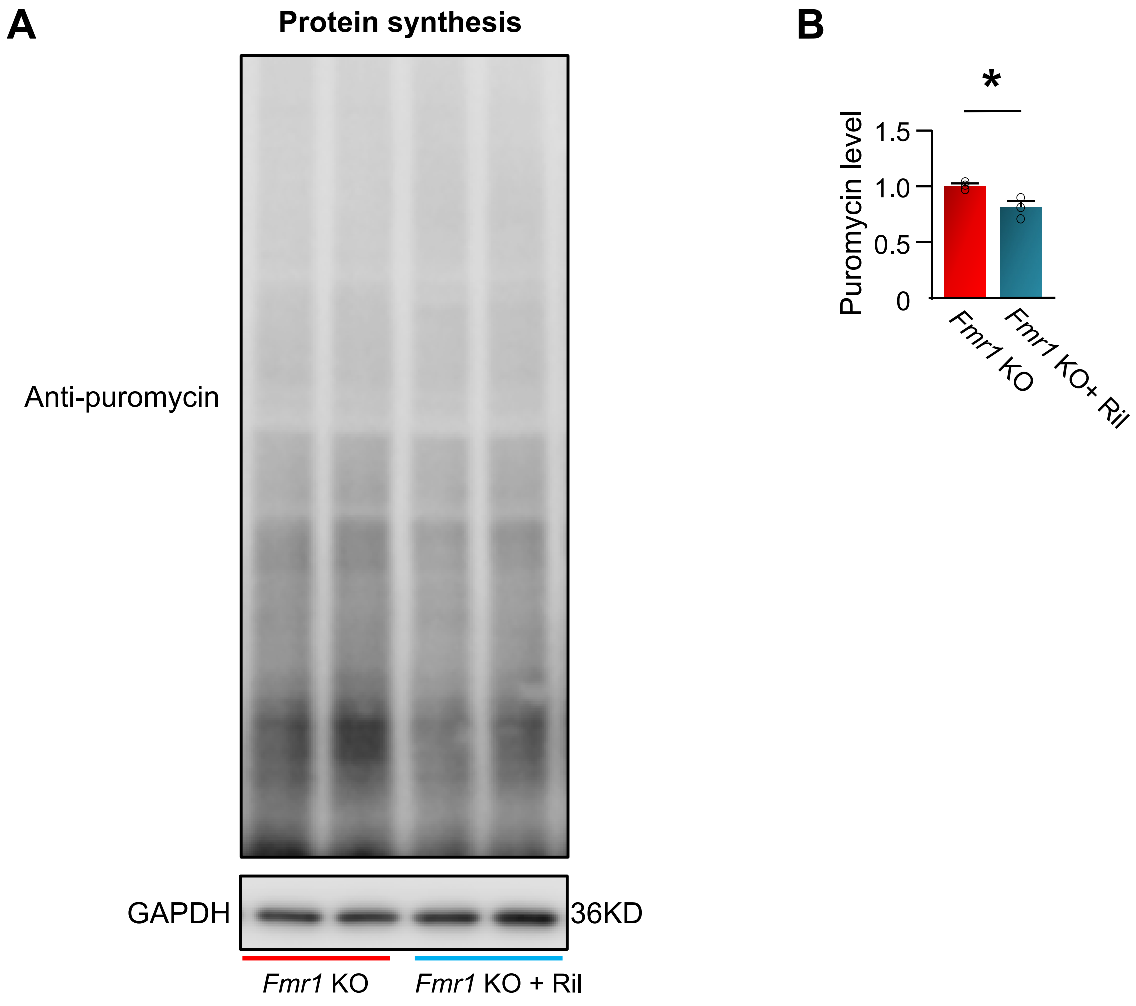

Supplement: Supplementary file 1 — Supplemental Materials [file 41380_2025_3207_MOESM1_ESM.pdf]
